# The Montecristo mining district, northern Chile: the relationship between vein-like magnetite-(apatite) and iron oxide-copper–gold deposits

**DOI:** 10.1007/s00126-023-01172-0

**Published:** 2023-03-28

**Authors:** Laura Mateo, Fernando Tornos, John M. Hanchar, Igor M. Villa, Holly J. Stein, Antonio Delgado

**Affiliations:** 1 Department of Earth Sciences, Memorial University of Newfoundland, St. John’s, NL A1B 3X5 Canada; 2 Instituto de Geociencias (CSIC-UCM), 28040 Madrid, Spain; 3 Institut Fürr Geologie, Universität Bern, 3012 Bern, Switzerland; 4 Centro Universitario Datazioni E Archeometria, Università Di Milano Bicocca, 20126 Milan, Italy; 5 Applied Isotope Research for Industry and Environment, AIRIE, Fort Collins, CO 80524 USA; 6 Department of Geosciences, University of Oslo, 0316 Oslo, Norway; 7 Laboratorio de Biogeoquímica de Isotopos Estables, Instituto Andaluz de Ciencias de La Tierra IACT (CSIC-UGR), 18100 Granada, Spain

**Keywords:** MtAp deposits, IOCG deposits, Mineral chemistry, Isotope geochemistry, Geochronology, Coastal Cordillera Andes, Chile

## Abstract

The Montecristo district, northern Chile, is one of the few places worldwide where there is a direct relationship between magnetite-(apatite) (MtAp) mineralization and iron oxide-copper–gold (IOCG) mineralization. The MtAp mineralization includes Ti-poor magnetite, fluorapatite, and actinolite and is crosscut and partially replaced by a younger IOCG mineralization that includes a second generation of actinolite and magnetite with quartz, chalcopyrite, pyrite, and molybdenite. The MtAp stage at Montecristo is interpreted as the crystallized iron-rich melts that used the pre-existing structures of the Atacama Fault System as conduits. These rocks later acted as a trap for hydrothermal IOCG mineralization. Geochronology data at Montecristo indicate that the host diorite (U–Pb zircon 153.3 ± 1.8 Ma, 2-sigma), MtAp mineralization (^40^Ar-^39^Ar in actinolite, 154 ± 2 Ma and 153 ± 4 Ma, 2-sigma), and the IOCG event (Re-Os on molybdenite, 151.8 ± 0.6 Ma, 2-sigma) are coeval within error and took place in a time span of less than 3.4 Ma. The εHf_i_ and εNd_i_ values of the host diorite are + 8.0 to + 9.8 and + 4.3 to + 5.4, respectively. The whole-rock ^87^Sr/^86^Sr_i_ values of the IOCG mineralization (0.70425 to 0.70442) are in the lower end of those of the MtAp mineralization (0.70426–0.70629). In contrast, εNd_i_ values for the IOCG mineralization (+ 5.4 and + 5.7) fall between those of the MtAp rocks (+ 6.6 to + 7.2) and the host diorite, which suggests that the IOCG event was related to fluids having a more crustal Nd (εNd_i_ <  + 5.7) composition than the MtAp mineralization. This likely reflects the mixing of Nd from the MtAp protolith and a deep magmatic-hydrothermal source, very likely an unexposed intrusion equivalent to the host diorite. Sulfur isotope compositions (δ^34^S, + 0.3 to + 3.4‰) are consistent with a magmatic source.

**Supplementary Information:**

The online version contains supplementary material available at 10.1007/s00126-023-01172-0.

## Introduction

Iron oxide-copper–gold (IOCG) and magnetite-(apatite) (MtAp) mineralization are a controversial group of ore deposits that share some common characteristics, such as the abundance of iron oxides and are associated with calcic-iron-alkali alteration. The largest deposit on Earth is the world-class Olympic Dam, which is thought to represent the IOCG-MtAp system as a whole (Hitzman et al. 1992). IOCG sensu stricto mineralization is widely interpreted as hydrothermal and consists of magnetite and/or hematite and chalcopyrite, with subordinate pyrite, pyrrhotite, and bornite, and accompanied by actinolite and alkali feldspar in deep environments and chlorite-sericite-feldspar at shallow depths. IOCG deposits occur from the Late Archean (e.g., Carajás district in Brazil) to the Mesozoic (e.g., Chilean and Peruvian belts) (Hitzman et al. 1992; Williams et al. 2005), and most of these deposits are replacive on and show a space–time association with felsic and mafic igneous rocks, from intrusive bodies to volcanic rocks (Williams et al. 2005; Skirrow 2022).

Two contrasting models have been proposed to explain IOCG mineralization: 1) a magmatic-hydrothermal origin related to the exsolution of fluids derived from crystallizing intrusions (Vila et al. 1996; Vivallo and Henríquez 1997, 1998; Pollard 2000; Haynes 2000; Marschik and Fontboté 2001b; Tristá and Kojima 2003; Sillitoe 2003; Williams et al. 2005; Pollard 2006; Groves et al. 2010; Tornos et al. 2010; Barton et al. 2011; Richards and Mumin 2013); or, 2) a hydrothermal fluid derived from the interaction of connate brines with evaporite deposits or fluids derived from them in convective cells (Hitzman 2000; Barton and Johnson 1996, 2000).

Magnetite-(apatite) (MtAp) mineralization, also referred to as Kiruna-type (Geijer 1931), or iron oxide apatite (IOA), are massive low-Ti (< 1 wt.% Ti) magnetite ores with variable amounts of apatite, actinolite or clinopyroxene (dominantly diopside), scapolite, and anhydrite (Badham and Morton 1976; Nyström and Henríquez 1994; Naslund et al. 2002; Valley et al. 2010; Day et al. 2016; Tornos et al. 2017; Liu et al. 2018; Heidarian et al. 2018), with many of them containing anomalously high concentrations of REEs and U associated with the apatite (Hitzman 2000; Naslund et al. 2000; Valley et al. 2010). A key diagnostic feature of MtAp systems is the presence of large crystals of apatite and actinolite, sometimes showing pegmatitic textures (Tornos et al. 2021). MtAp deposits are known to occur from the Early Proterozoic (e.g., Kiruna, Sweden) (Geijer 1931) to the Pliocene (El Laco, Chile) (Park 1961), and are principally associated with calc-alkaline to alkaline igneous rocks (Naslund et al. 2000). They also include a pervasive metasomatic alteration of the host rocks similar to that of IOCG systems, with large zones of calcic-iron-alkali alteration (Hitzman 2000) that may grade upwards into hydrolytic alteration (Hitzman et al. 1992).

Different hypotheses have been proposed for the origin of MtAp deposits that include: 1) crystallization from an Fe-rich melt that separates from a more felsic melt (Badham and Morton 1976; Henríquez and Martin 1978; Frietsch 1978; Philpotts 1981, 1982; Nyström and Henríquez 1994; Naslund et al. 2002; Lledó and Jenkins 2008; Velasco et al. 2016; Nyström et al. 2016; Hou et al. 2018; Lledó et al. 2020); the crystallization is in many locations coeval with the exsolution of large amounts of hydrothermal fluids (Tornos et al. 2016, 2017); 2) circulation of hydrothermal fluids derived from the crystallization of mafic silicate melts that lead to the complete replacement of pre-existing rocks (usually andesite) by massive magnetite (Hildebrand 1986; Ménard 1995; Rhodes et al. 1999; Sillitoe and Burrows 2002; Gandhi 2003; Corriveau et al. 2016); 3) replacement of pre-existing rocks by heated hydrothermal fluids of non-magmatic origin such as basinal brines, or non-magmatic fluids equilibrated with evaporites (Hitzman 2000; Barton and Johnson 1996, 2000; Rhodes and Oreskes 1999); 4) the crystallization of magnetite microlites from a silicate melt followed by buoyant segregation from crystallizing mafic rocks, and flotation of the magmatic magnetite-bubble pairs, deposition of massive magnetite along faults, and posterior growth of hydrothermal magnetite (Knipping et al. 2015a); and, 5) crystallization from a sulfate and iron-rich magma being the product of the melting of shallow marine sediments by intruding andesite (Bain et al. 2020, 2021).

The relationship between both styles of mineralization remains a controversial topic. Early works (Hitzman et al 1992; Sillitoe 2003) included magnetite-(apatite) as part of the same system, while others have proposed that are part of the same clan (Williams et al. 2005) or end members on a continuous spectrum of mineralization (Hitzman et al. 1992; Vivallo and Henríquez 1997; Hitzman 2000; Gandhi 2003; Corriveau et al. 2016; Simon et al. 2018; del Real et al. 2021). This interpretation is based on the shared characteristics of IOCG and MtAp deposits, such as the similar mineral assemblage, related metasomatic alteration, and the local spatial association. This relationship is reinforced by the local presence of sparse hydrothermal late-stage Cu-Fe sulfides and gold overprinting some MtAp deposits or in their vicinity (e.g., Marcona in Peru, Chen et al. 2010a; and Cerro Negro Norte in Chile, Vivallo et al. 1995, Salazar et al. 2019), or the presence of mineralization ascribed to the IOCG group near clusters of MtAp deposits such as in the Norrbotten district, Sweden (Martinsson et al. 2016; Bauer et al. 2021) or the Great Bear Magmatic Zone, Canada (Ootes et al. 2017). However, on a global scale, MtAp systems are much more abundant than IOCG systems, and only locally do they coexist, such as in Carajás in Brazil (Xavier et al. 2012; Schutesky and de Oliveira 2020), Olympic Dam in Australia (Ehrig et al. 2012), or the Coastal Cordillera of the Andes (Espinoza 1990; Sillitoe 2003).

Other studies have suggested that MtAp and IOCG systems are genetically unconnected and form different ore systems, although they are sometimes superimposed. The detailed structural study of Bauer et al. (2018, 2021) in the Norrbotten region shows that in many cases, the IOCG mineralization can be significantly younger and unrelated to the MtAp mineralization, and the massive magnetite is just a chemical trap for the later Cu-Au-rich event.

In the Coastal Cordillera of the Andes, MtAp systems are dominantly vein-like, up to 50–100 m thick, with only a few extrusive tops, despite that locally, some stratabound replacive orebodies form when the veins crosscut rocks that are favorable for fluid-rock interactions (Espinoza 1990; Henríquez et al. 1994; Travisany et al. 1995; Tornos et al. 2021). Most well-known IOCG deposits in the Coastal Cordillera of the Andes are replacive in volcanoclastic andesite (e.g., Mina Justa, Peru, Chen et al. 2010b; Punta del Cobre district, Chile, Marschik and Fontboté 2001b, Arévalo et al. 2006, Del Real et al 2021; Dominga, Chile, Arredondo et al. 2017). However, in the northern part of the Coastal Cordillera in Chile, some smaller deposits occur as subvertical MtAp and IOCG veins in the plutonic rocks underlying the Jurassic-Cretaceous mafic volcanics such as the Montecristo, Tocopilla and Gatico districts (Boric et al. 1990; Sillitoe 2003). In these plutonic-hosted veins, Espinoza (1990) and Sillitoe (2003) have proposed a vertical evolution from deep MtAp to shallower IOCG mineralization both being channelized by large trans-crustal faults that also host diorite. A similar zonation model has been expanded by Simon et al. (2018) to the giant IOCG deposits of Mantoverde and Punta del Cobre in Chile. At the dominantly stratabound deposits in the Punta del Cobre district (which includes the Punta del Cobre and Candelaria deposits, among others), there are large bodies of apatite-poor massive magnetite and only sparse veins that could be unambiguously assigned to the MtAp style mineralization (Arévalo et al. 2006; Marschik and Fontboté. 2001b; Del Real et al. 2018; J. Carriedo, pers. com., 2020).

In the Carmen-Sierra Áspera district, which is very similar to Mantoverde (located ~ 40 km to the southeast), U–Pb dating of hematite in the Carmen de Cobre deposit suggests that the IOCG mineralization is *ca*. 10 My younger than the MtAp rocks of the Carmen de Fierro deposit (located ~ 5 km north of Carmen de Cobre) (Gelcich et al. 2005; Verdugo-Ihl et al. 2022). In the Marcona-Mina Justa district in western Peru, the MtAp ore and the related diopside-apatite pegmatite are at least 20 My older than the associated IOCG mineralization (Chen et al. 2011; Tornos et al. 2023) confirming that there is not a continuum between both styles of mineralization.

Therefore, one of the few places for elucidating the geochemical and geochronological relationship between unambiguous MtAp and IOCG systems are the vein-type deposits such as the Montecristo and Tocopilla districts, northern Chile, where the veins include both styles of mineralization. These veins represent a larger group of veins extending from northern Chile into southern Peru (Sillitoe 2003; Tornos et al. 2021). The absence of stratabound or shear-controlled replacive mineralization minimizes the effects of fluid-rock interaction in the mineralogy and geochemistry of the ores.

In the present study, the results from a detailed mineralogical, geochemical, and geochronological investigation of selected veins in the Montecristo district reveal that although the IOCG and MtAp deposits are in close proximity, and are broadly coeval, the geochemical data suggests that perhaps they are not part of the same ore forming system.

## Geological background

The Andean magmatic arc started in the Jurassic as a consequence of the subduction of the oceanic lithosphere in the westernmost margin of the South American plate after the breakup of Gondwana, and it extended along the present-day Coastal Cordillera till nowadays (Dalziel et al. 1987; Mpodozis and Ramos 1990; Charrier et al. 2007). The oblique subduction led to the formation of a large strike-slip structure, the Atacama Fault System (AFS), which has a dominant sinistral component and extends along the Coastal Cordillera from ~ 20°30’ to ~ 29°45’S latitude (Hervé 1987; Mpodozis and Ramos 1990; Scheuber and Reutter 1992; Brown et al. 1993; Cembrano et al. 2005). North of the Bolivian orocline (Capitanio et al. 2011), equivalent structures have a WNW-ESE trend.

During the Late Jurassic-Early Cretaceous, the AFS experienced alternating both transtensional and transpressional movement (Cembrano et al. 1997, 2005; Grocott and Wilson 1997). These structures controlled the emplacement of the plutonic rocks and metasomatic alteration by hydrothermal fluids in the area (Grocott and Wilson 1997).

The Coastal Iron Belt (CIB) is situated along the Coastal Cordillera between 12º and 31ºS latitude and includes most of the IOCG and MtAp deposits in northern Chile and southern Peru, among others, such as porphyry copper deposits, Cu and Au-bearing veins, and “manto-type” copper deposits (Vivallo et al. 2000; Maksaev et al. 2007). The CIB is hosted by up to a 10-km thick regionally extensive sequence of Jurassic and Early Cretaceous subaerial basaltic andesite to andesite of the La Negra Formation and equivalent lithologies in southern Peru, represented by the Rio Grande Formation (Jaillard et al. 2000). Stratigraphically equivalent is the subaqueous Punta del Cobre Formation to the south, interpreted as deposited in an intra- to back-arc setting (Marschik and Fontboté 2001a). The La Negra Formation rocks have predominantly high-K to calc-alkaline affinities (Pichowiak et al. (1990); however, rocks of tholeiitic compositions extruded during the initial stages of the magmatic arc also exist (Pichowiak et al. 1990; Lucassen and Franz 1994). The volcanic sequence is intruded by Late Jurassic to Early Cretaceous plutonic rocks with similar geochemistry (Espinoza 1990). Together, they represent the magmatism that marks the onset of the Andean arc.

The Atacama Fault System has played a major role in the location of the IOCG and MtAp deposits (Boric et al. 1990; Freraut and Cuadra 1994; Vila et al. 1996; Gelcich et al. 1998; Sillitoe 2003; Vivallo et al. 2008; Tornos et al. 2021). Most of the MtAp mineralization is located on the main AFS, but IOCG mineralization is also related to N-S and NW–SE subsidiary structures, with both sinistral strike-slip and normal dip-slip extensional movements (Dallmeyer et al. 1996; Cembrano et al. 2005).

Systematic K–Ar and ^40^Ar-^39^Ar dating of hydrothermal silicates (e.g., actinolite, K-feldspar, biotite, white mica) indicate that most IOCG and MtAp deposits in the CIB were formed during two events of Middle to Late Jurassic (~ 170–150 Ma) and Early Cretaceous (~ 130–110 Ma) age, but there is also some IOCG mineralization of likely late Cretaceous and even Paleocene age (Boric et al. 1990; Vila et al. 1996; Vivallo and Henríquez 1998; Gelcich et al. 1998, 2003; Sillitoe 2003; Vivallo et al. 2008; Arredondo et al. 2017) which suggests that the ore forming events span more than 130 Ma.

## The Montecristo district

**Fig. 1:**
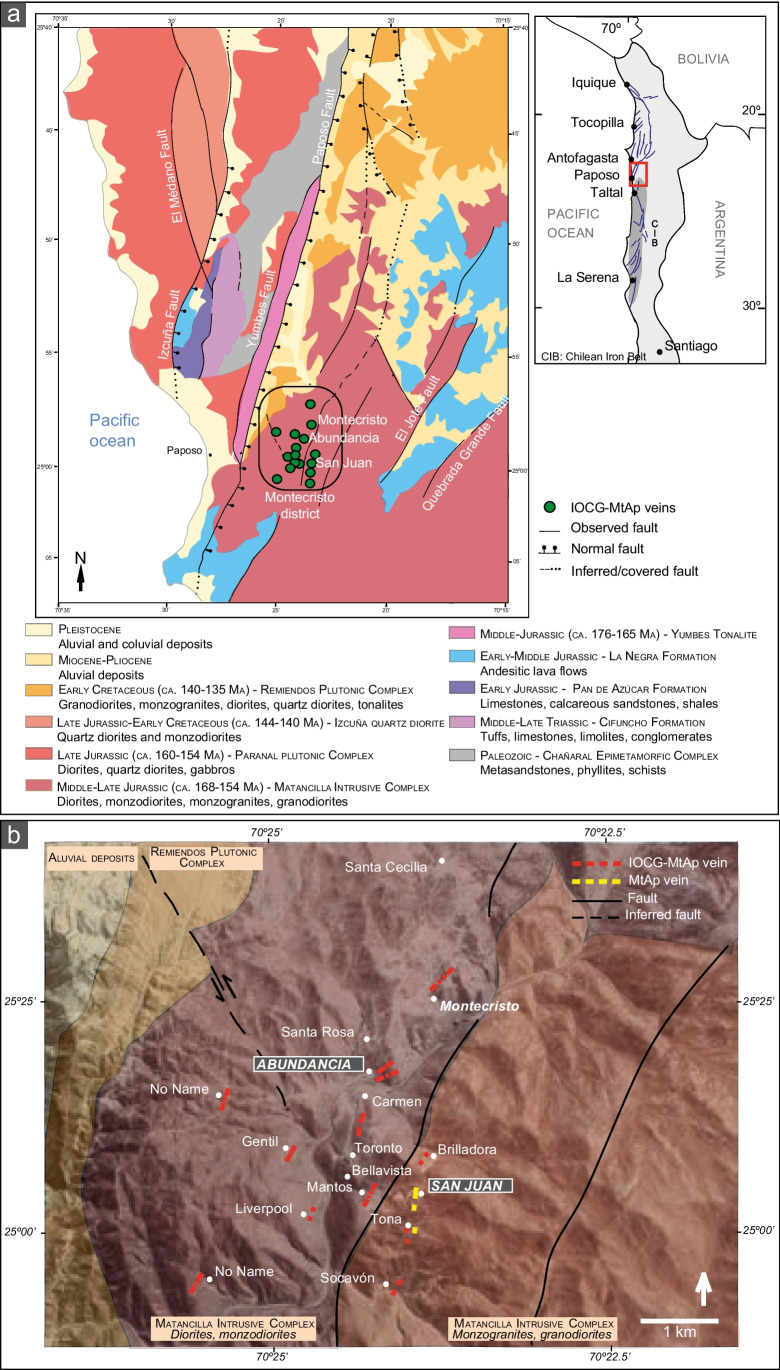
(a) Location map and simplified regional geology of the Montecristo district (modified from Escribano et al. 2013 and Álvarez et al. 2016). (b**)** Location of the IOCG and MtAp veins and simplified geological map of the Montecristo district (modified from Álvarez et al. 2016)

The Montecristo mining district, the focus of the present study, is located in the westernmost Middle-Late Jurassic sub-belt of the CIB (Espinoza et al. 1996; Vivallo and Henríquez 1998; Sillitoe 2003) (Fig. 1a) where the AFS separates volcanic rocks to the east from plutonic rocks to the west. The mineralized veins in this district are hosted by the Matancilla Intrusive Complex (MIC) (Naranjo and Puig 1984; Escribano et al. 2013; Álvarez et al. 2016; Fig. 1a). The Complex includes multiple lithologies, including granodiorite, diorite, quartz diorite, and monzogranite and have been dated between 178 to 154 Ma (Mavor et al. 2022). Overall, these plutonic rocks are metaluminous and have tholeiitic to calc-alkaline, to high-K affinities (Álvarez et al. 2016). In detail, the host rock to the mineralization is a medium grained, inequigranular and unoriented amphibole-rich diorite.

The Montecristo district hosts at least twelve mineralized veins striking roughly NNE-SSW that are ~ 500 to ~ 5,000 m long and ~ 3 to ~ 30 m wide (Fig. 1b; Boric et al. 1990). The IOCG mineralization includes dominant magnetite and actinolite with lesser amounts of quartz, chalcopyrite, pyrite, bornite, molybdenite, and minor titanite. The ore includes small quantities of gold, and some veins show a geochemical anomaly enriched in Ag, Pb, Zn, and V (Espinoza et al. 1996). There is a large oxidation zone capping the vein and includes atacamite, chrysocolla, antlerite, and minor stringhamite [CaCuSiO_4_•2(H_2_O)] and chenevixite [Cu_2_Fe^3+^_2_(AsO_4_)2(OH)_4_]. A minor “cementation zone” between the oxidized and primary zones is composed of covellite and chalcocite (Espinoza et al. 1996).

The mineralized veins in the Montecristo district are located in the Paposo segment, one of the three parts of the AFS between Antofagasta and Paposo (Brown et al. 1993). In detail, they are structurally controlled by tensional faults oblique to the main AFS and formed during a sinistral strike slip event (Tornos et al. 2021).

The best-known deposit in this district, the Montecristo vein, is a magnetite-rich vein striking N45º-50ºE and dipping 75-80ºNW, with an average width of ~ 10 m but at some places reaching a thickness of ~ 30 m. The recognized vertical extension is about 400 m. The vein was discovered in 1850; since then, ~ 10 Mt ore has been mined. Despite the economic importance of the Montecristo vein, this study focuses on the currently mined Abundancia vein and the cropping out but unmined San Juan vein due to its key geological characteristics. The Abundancia vein contains both MtAp and IOCG mineralization and has been historically an important copper source in the region. The San Juan vein is different from the other veins in the Montecristo district since it is a classical MtAp vein composed of magnetite, apatite, and actinolite with no IOCG-like overprint.

The host diorite has been irregularly altered by hydrothermal fluids to an unknown extent. The original plagioclase is in some locations replaced from the center to the edge of the grains by potassium feldspar; this alteration is followed by a later, low temperature, pervasive replacement by adularia, albite, or white mica, and local late calcite. Magmatic amphibole has been strongly chloritized (ESM Fig. 1). The immediate halo of the veins shows a texturally destructive alteration up to some cm-thick that includes albite, quartz, magnetite, chlorite, actinolite and white mica (Vivallo and Henríquez 1998). The halo of pervasive alteration seems to increase in size and intensity downwards.

## The Abundancia MtAp-IOCG vein

**Fig. 2:**
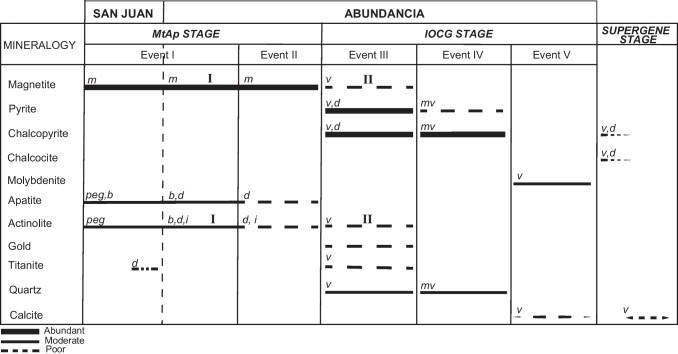
Mineral assemblages of the MtAp and the IOCG mineralization stages in the San Juan and Abundancia veins. Mineral textures and structures: b: banded; d: disseminated; i: inclusions; m: massive; mv: massive veins; peg: pegmatite; v: veinlets

**Fig. 3:**
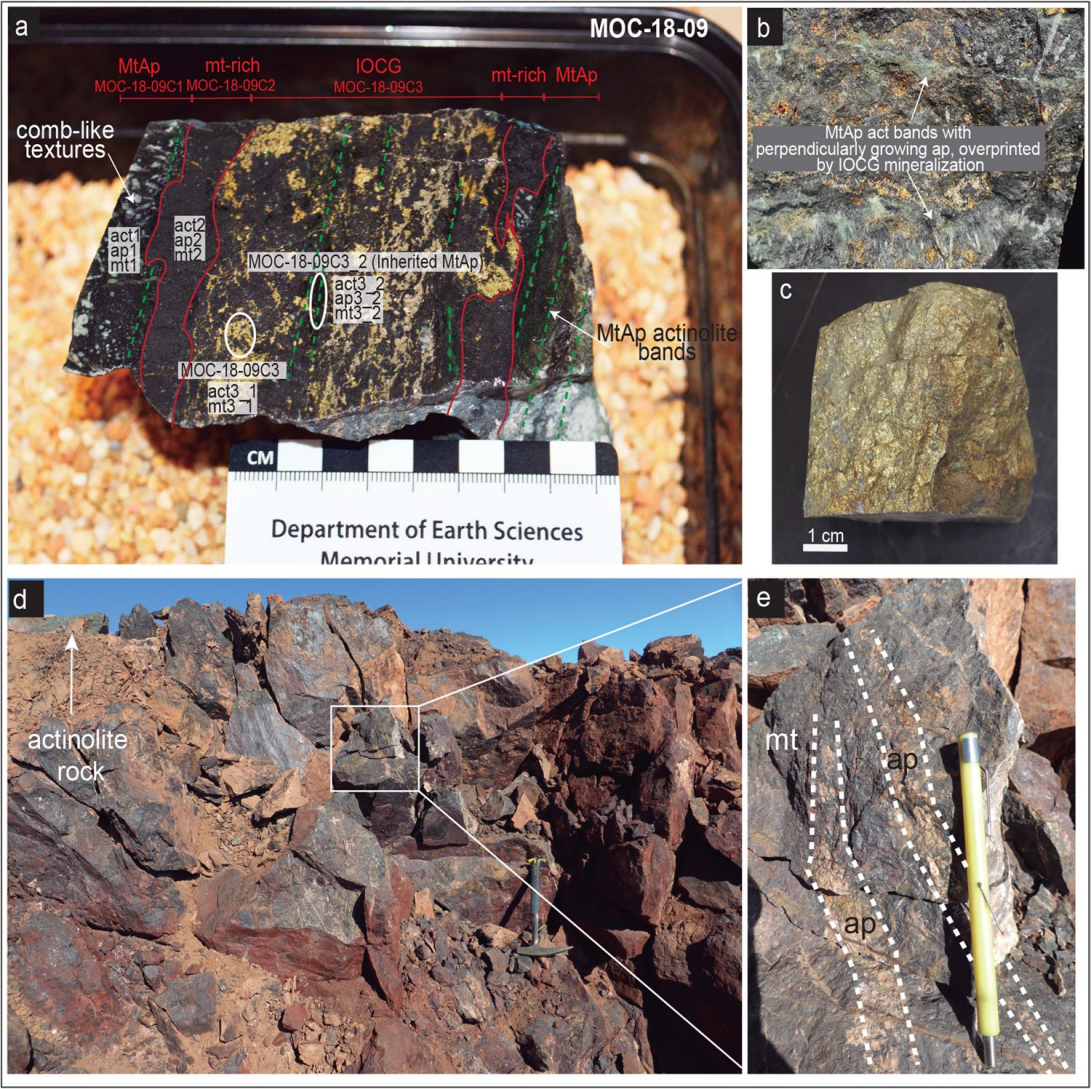
(a) Mineralized sample showing the MtAp and IOCG events in the deposit (sample MOC-18–09). (b) Banded MtAp actinolite with perpendicular growth of coarse-grained apatite and magnetite overprinted by the IOCG (sample MOC-18–09). (c) Massive chalcopyrite vein with quartz in Abundancia (sample MOC-18-11a). Samples from the Abundancia vein. (d) The San Juan MtAp vein, with coarse-grained actinolite rocks at the back. (e) Magnetite from San Juan with banded coarse-grained apatite

The Abundancia vein is a N40ºE and ~ 4 m width copper-rich (1.4% Cu) vein, with subordinate amounts of gold (0.4–0.5 g/t) and molybdenum (0.03%), and small concentrations of Co and Ni, which has been mined down to 940 m below the land surface. Several hypogene mineral assemblages are recognized, which are part of the following assemblages (Fig. 2): 1) an early MtAp assemblage (MtAp I) that includes magnetite, apatite, and actinolite predominantly arranged as cm-thick vertical subparallel bands. The pale green to dark green actinolite and the centimeter-sized crystals of white apatite can have comb-like unidirectional growth textures (Fig. 3a, b & e) growing perpendicular to the edge of the veins (Fig. 3a & b); 2) A second MtAp event (MtAp II) having the same mineral assemblage but with decreasing grain size and abundant massive magnetite occurring as crosscutting veins (Fig. 3a); 3) an IOCG stage that seems to dominate in the upper parts of the Abundancia vein and clearly crosscut and replaces the MtAp assemblages (Fig. 3a & b). Minerals in this stage include medium-grained magnetite, dark green actinolite, chalcopyrite, pyrite, quartz, titanite and gold; pyrite is replaced by later chalcopyrite; 4) a copper-rich event with up to ~ 20 cm thick massive veins with chalcopyrite and quartz (Fig. 3c); and 5) a late stage event with small veinlets of calcite or laminar molybdenite along fault surfaces, which cut both the earlier MtAp and IOCG mineralization.

In the deepest levels of the system (~ 940 to ~ 915 m), there is a brecciated monzogabbro which is partially replaced by an andradite-diopside-magnetite assemblage coeval with a halo of external albitization and has a retrograde replacement of pyroxene by actinolite and veinlets of actinolite and hydroxyapatite, with minor biotite, quartz, chalcopyrite, and pyrite (Díaz et al. 2018).

## The San Juan MtAp vein

The San Juan MtAp vein, located approximately 1.5 km south of the Abundancia vein, has a NE-SW strike, ~ 4 m width, and ~ 600 m of inferred length. It contains massive magnetite with subvertical bands variably enriched in coarse-grained fluorapatite and actinolite (Fig. 2) that form pegmatite-like textures similar to those found in the Abundancia vein (Fig. 3d & e). Outcrops of this vein show a strong supergene alteration with replacement of the magnetite by hematite and late cross-cutting calcite veinlets.

## Analytical methods

### Petrography

Samples for petrographic studies were selected from the Abundancia and San Juan veins. Polished thin sections from the mineralization and the host rock were used for transmitted and reflected light microscopy, and for cathodoluminescence (CL) imaging on apatite to reveal the distribution and metasomatic alteration. Back-scattered electron (BSE) imaging was done on two 25 mm diameter, by 6 mm tall epoxy mounts containing several rock billets after polishing and carbon coating. The billets were examined with a JEOL 7100F field emission gun scanning electron microscope (FEG-SEM) at Memorial University of Newfoundland equipped with energy-dispersive spectroscopy (EDS) at 15 kV and 50 nA. The same epoxy mounts were used for further analyses by Electron Probe Microanalysis (EPMA) and Laser Ablation Inductively Coupled Plasma Mass Spectrometry (LA-ICPMS).

### Electron probe microanalysis (EPMA)

The electron probe microanalyzer (EPMA) analyses were done using a JEOL JXA-8230 SuperProbe at Memorial University of Newfoundland. Details for the EPMA analyses are summarized in the ESM Table 2A for actinolite analyses, ESM Table 2C for magnetite, and ESM Table 2E, for apatite.

The EPMA was also used to produce X-ray maps of actinolite in the same areas where the traverses were done and analyzed for Ti, Ca, Fe, Mg, and K based on previous determinations of the actinolite chemical composition. Potassium was included in order to determine its distribution for later ^40^Ar-^39^Ar dating. For each X-ray map, an accelerating voltage of 15 kV, a sample current of 200 nA, and a pixel dwell time of 100 ms were used.

**Fig. 4:**
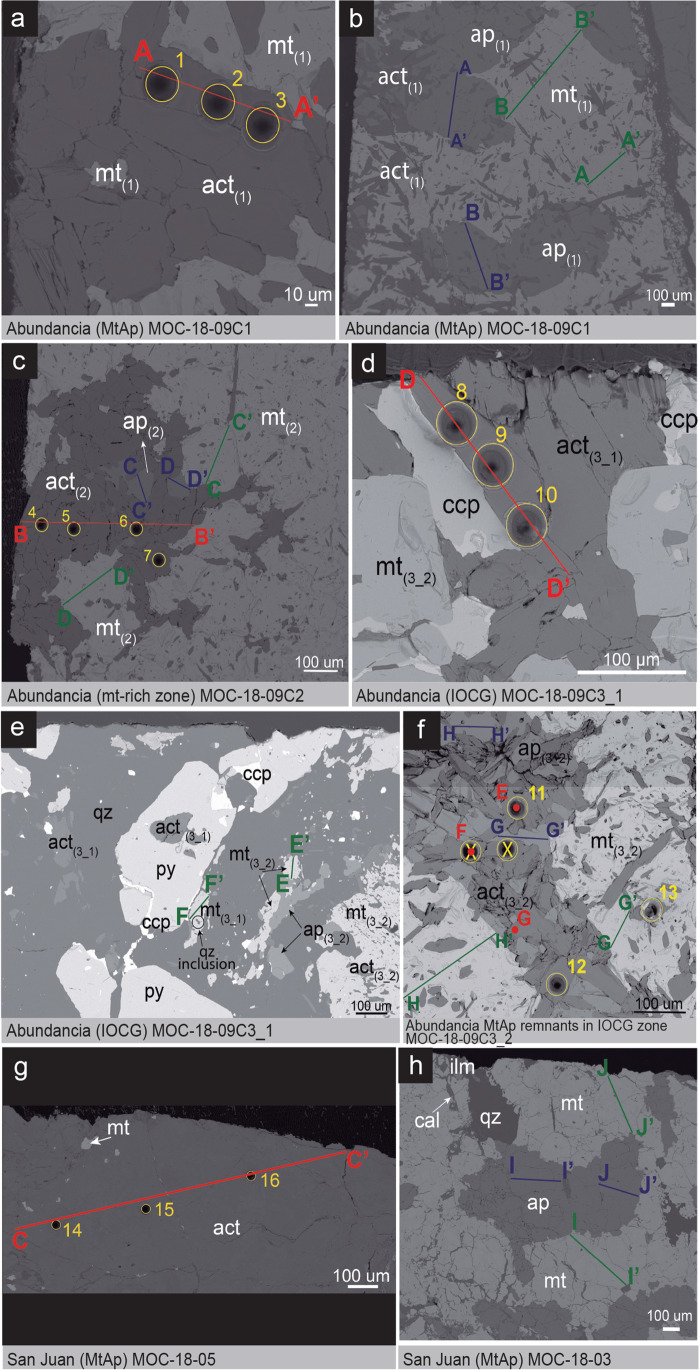
BSE images showing EPMA analyses done on actinolite (red), magnetite (green), and apatite (blue), and the spot location of the LA-ICPMS trace elements analyses done on actinolite grains (yellow). (a) & (b) MtAp I mineralization in the Abundancia vein. (c) MtAp II mineralization in the Abundancia vein. (d) & (e) IOCG mineralization in the Abundancia vein. (f) Remnants of the MtAp mineralization in the IOCG zone. (g) & (h) Coarse-grained actinolite, and magnetite and apatite rock from the San Juan vein

EPMA traverses and spot analyses were done on representative grains of actinolite, magnetite, and apatite for major and minor elements (Fig. 4). Analyses include Ti, Mn, K, Ca, S, Na, Al, Si, Mg, Fe, F, and Cl for the actinolite. Special attention was devoted to the analysis of potassium, which as mentioned above is critical for selecting samples suitable for later ^40^Ar-^39^Ar geochronology (see below). The magnetite EPMA analyses included Cu, Ni, Mn, Ti, V, K, Ca, S, P, Na, Al, Si, Mg, Zn, Co, Fe, Cr, and Ba, and apatite was analyzed for F, Fe, Mn, As, Ca, Sr, S, Na, Si, Mg, Cl, Y, and P. EPMA traverses were done across mineral grains avoiding inclusions, cracks, and obvious alteration zones.

### LA-ICPMS trace elements of actinolite

The same epoxy mounts containing actinolite from the Abundancia and the San Juan veins were used for in situ laser ablation inductively coupled plasma-mass spectrometry (LA-ICPMS) trace elements analyses (Fig. 4a, c, d, f & g). The analyses were done on either the traverse or the spots where the EPMA analyses were previously done. A total of 43 trace elements were measured by LA-ICPMS, shown in the ESM Table 2A.

The actinolite trace element analyses were done at the Micro Analysis Facility (MAF) at Memorial University of Newfoundland, using a Thermo Finnigan Element XR high-resolution (HR), double-focusing magnetic sector-inductively coupled-plasma mass spectrometer (HR-ICPMS), coupled to a GeoLas 193-nm Excimer laser system. Details for the LA-ICPMS analyses are summarized in the ESM Table 2A. The Ca concentration in the actinolite determined by EPMA was used as the internal standard for calibrating each LA-ICPMS analysis. Analyses were done using a pulse frequency of 10 Hz, an energy density of 4 J/cm^2^, and a 40 µm diameter spot size. For each analysis, the background was measured for around 30 s, followed by 60 s of laser ablation. NIST 610 was used as the primary standard, and USGS BCR-2G and NIST 612 were used as secondary standards and were interspersed during the trace element analyses.

### ^*40*^*Ar/*^*39*^*Ar geochronology in actinolite*

The study includes two ^40^Ar/^39^Ar dates of actinolite, one fine-grained actinolite associated with the MtAp I event in the Abundancia vein (sample MOC-18–09), and one coarse-grained actinolite with moderate chloritization from the San Juan vein (sample MOC-18–05). Actinolite grains were crushed using a Plattner mortar-and-pestle; individual fragments were handpicked under a binocular microscope using tweezers, avoiding altered fragments and impurities, such as calcite. Both samples were subsequently crushed again using a Plattner mortar-and-pestle and sieved to a size of 90–150 µm to obtain a concentrate of pure actinolite.

Sample separates (15 and 18 mg, ESM Table 4) were irradiated in the McMaster University nuclear reactor (Ontario, Canada) for 30 MWh, carefully avoiding Cd shielding. The age monitor was hornblende MMhb1 (McClure Mountain Complex, Colorado) with an assumed age of 523.98 Ma (Schoene and Bowring 2006). ^40^Ar/^39^Ar analyses by step-heating following the procedures described by Bosio et al. (2020) were done on the Nu Instruments Noblesse noble gas mass spectrometer at the University of Milano-Bicocca, Milan, Italy, equipped with one Faraday cup with a 10^11^ Ω resistor and two ion counters. Before each sample was analyzed, a blank measurement was followed by two successive measurements of atmospheric Ar delivered by a pipette system. The analytical protocol consisted of four measurement cycles to allow an in-run cross-calibration of the relative collector gains, F/IC0 and IC0/IC1, in each individual run under the (variable) mass spectrometer conditions. The four cycles were: ^40^Ar (F), ^38^Ar (IC0), ^36^Ar (IC1); ^39^Ar (F), ^37^Ar (IC0), ^35^Cl (IC1); ^41^C_3_H_5_ (F), ^39^Ar (IC0), ^37^Ar (IC1); ^38^Ar (F), ^36^Ar (IC0). Each measurement consisted of 25 repetitions of the four cycles to avoid artefacts due to ion counter nonlinearities (Barberini and Villa 2015). The ion counter gains are similar, but not necessarily equal, to those determined from the atmospheric pipettes (which also independently quantify the mass fractionation originating in the source). The raw data from the mass spectrometer zero-time extrapolation were processed with an in-house Excel spreadsheet by correcting for mass spectrometer background, ion counter gains, blank measurements, source fractionation, and decay of ^37^Ar since irradiation, as well as propagating all the associated uncertainties. The total concentrations of ^39^Ar, ^38^Ar, and ^37^Ar were converted to absolute concentrations of K, Cl, and Ca, respectively, to control stoichiometry; and provide the Ca/K and Cl/K ratios. Since the stoichiometry of the present samples was determined by EPMA, for the age calculation, we consider only “isochemical steps” (Villa et al. 2006) with constant Ca/K and Cl/K ratios matching the EPMA measurements. The ^40^ K decay constant used for the age calculation was 5.543 × 10^–4^ Ma^−1^ (Steiger and Jäger 1977), even if it is very probably slightly inaccurate (Min et al. 2000; Naumenko-Dèzes et al. 2018) was used, by convention, in order to make intercomparisons with literature data less cumbersome. Different levels of uncertainty must also be considered. The internal concordance/discordance of step ages is assessed based on in-run uncertainties only. The inter comparison of samples analyzed in one, and the same laboratory requires compounding the in-run uncertainty with that on the neutron flux gradient. Finally, the inter comparison of the present samples with those reported in the literature requires inclusion of the systematic uncertainties on the age of the irradiation monitor (if the information is provided at all) and of the uncertainty of the ^40^ K decay constant, where necessary.

### LASS U–Pb geochronology and Hf tracer isotopes on zircon

Representative zircon crystals from the Abundancia host diorite (sample MOC-18–02) were analyzed in situ for U–Pb, and Lu–Hf isotopes. The diorite was processed using a jaw crusher, and a dish mill afterwards, retaining > 90% of the crushed material between < 500 µm and > 63 µm. Magnetic separation and heavy liquids, bromoform and methylene iodide, separated and concentrated the zircon from the other minerals. The zircon grains were handpicked under the binocular microscope and mounted in epoxy. The mounts were polished to expose the crystal centers and carbon coated. Cathodoluminescence images were obtained using a JEOL 7100F field emission gun scanning electron microscope (FEG-SEM) at Memorial University of Newfoundland equipped with energy-dispersive spectroscopy (EDS) at 15 kV and 50 nA. These images were acquired in order to select the best zircon grains and areas for the in situ analyses, avoiding inclusions of other minerals, inherited cores, or cracks.

Lu–Hf isotopes paired with U–Pb isotopes were analyzed by laser ablation split stream (LASS) at the Micro Analysis Facility at Memorial University of Newfoundland. We followed the instrument configuration, operating parameters, and data reduction methods outlined by Fisher et al. (2011; 2014a, b) with the exception that N_2_ was added to the Ar carrier gas to both mass spectrometers for increased sensitivity.

The Lu–Hf analyses were done using a Thermo Finnigan Neptune multi-collector inductively coupled plasma mass spectrometer (MC-ICPMS). The U–Pb analyses were done simultaneously using a Thermo Finnigan Element XR high-resolution magnetic-sector inductively coupled plasma mass spectrometer (HR-ICPMS). Both mass spectrometers were interfaced to a GeoLas Pro 193 nm Ar-F excimer laser, operating at 10 Hz, 5 J/cm^2^, and a 40 μm diameter spot size. After collecting a background for 30 s, the samples were ablated for 60 s. The data were reduced using Iolite (Paton et al. 2011).

The Lu–Hf analyses done on the Montecristo MOC-18–02 zircon crystals were interspersed with analyses of several zircon reference materials to assess accuracy, mass bias corrections, and external reproducibility. While all of these reference materials listed below were analyzed during the LASS analyses, not all the reference materials were used for both the Hf-Lu and U–Pb data reduction.

The zircon reference materials used in this study for the Lu–Hf LASS analyses (determined in previous studies by solution MC-ICP-MS) covered the range of (Lu + Yb)/Hf of the MOC-18–02 zircon crystals included MUNZirc-1 (B-140) (^176^Hf/^177^Hf = 0.282135 ± 7; Fisher et al. 2011), MUNZirc-4 (B-144) (^176^Hf/^177^Hf = 0.282135 ± 7; Fisher et al. 2011), Plešovice (^176^Hf/^177^Hf = 0.282482 ± 13; Sláma et al. 2008), FC-1 (^176^Hf/^177^Hf = 0.282182 ± 14; Fisher et al. 2014a, b); and R33 (^176^Hf/^177^Hf = 0.282767 ± 18; Fisher et al. 2014a, b).

The zircon reference materials used in this study for the U–Pb LASS analyses included 91,500 (1065 Ma, Wiedenbeck et al. 2004), which was used as the primary U–Pb reference material, and Plešovice (337.13 Ma, Sláma et al. 2008); FC-1 (1099 Ma, Paces and Miller 1993), R-33 (419 Ma, Black et al. 2004); Temora 2 (417 Ma, Black et al. 2004), and 02,123 (295 Ma, Ketchum et al. 2001) were used as secondary reference materials.

Initial εHf values were calculated using a present-day CHUR value of ^176^Hf/^177^Hf = 0.282785, ^176^Lu/^177^Hf = 0.0336 as reported by Bouvier et al. (2008) and the ^176^Lu decay constant of 1.867 × 10^–11^ yr^–1^ from Söderlund et al. (2004).

The ^238^U counts in the zircon grains from sample MOC-18–02 were anomalously high in the first batch of analyses (e.g., individual analyses were done on 46 grains) due to the high U concentrations in the zircon (e.g., 920–7,330 ppm). The in situ U–Pb analyses showed significant discordance caused by Pb loss, radiation damage, or a combination of both factors. A second batch of zircon grains (27 grains, one analysis per grain) also from sample MOC-18–02 were chemically abraded, generally following the method in Mattinson (2005) prior to the LASS analyses for U–Pb and Hf isotopes. The zircon grains were annealed for 48 h at 1100 °C in the air in a fused silica crucible. This was followed by partial dissolution in concentrated (i.e., 49%) HF sealed in a Parr Bomb in an oven at 190 °C for 2 h (the Parr Bomb was put into an oven at pre-set at 190 °C, and then after two hours at 190 °C the Parr Bomb was removed from the oven and allowed to cool for one hour to room temperature before opening). This procedure removes radiation-damaged, altered, or metamict zones in zircon that cannot be restored with annealing, thus virtually eliminating secondary lead loss. This method often results in, for simple grains of one age, concordant or near-concordant analyses.

Preliminary U–Pb data after annealing, however, produced similar results to the first batch of LASS analyses, likely because the uranium content in the zircon grains was still anomalously high (e.g., 560 to 5,320 ppm). Thus, the zircon accumulated radiation damage dose (i.e., alpha decay events per milligram of zircon) was calculated for each zircon using the equation from Murakami et al. (1991) to eliminate the extensive radiation-damaged zircon grains, or regions in zircon grains, in an attempt to get a concordant age. After the radiation damage calculations, zircon grains with radiation damage in stages 2 and 3 (Nasdala et al. 2004 modified the damage stages initially reported by Murakami et al. in 1991) were removed, keeping the 12 zircon grains in stage 1 that were > 90% concordant for the final age calculation and used for the εHf calculation.

### Re-Os geochronology in molybdenite

Molybdenite (MoS_2_) has very high Re/Os during crystallization; this mineral incorporates almost no Os (Stein et al. 2001). Therefore, ^187^Re-^187^Os dating of molybdenite uses the following simplified equation: ^187^Os_measured_ = ^187^Re_measured_ × (e^λt^—1), where λ is the decay constant for ^187^Re (1.666 × 10^–11^ yr^–1^; Smoliar et al. 1996).

Re-Os isotopic analyses were done on two molybdenite samples from level 940 (m) of the Abundancia vein. There is no evidence of supergene alteration, since the petrography shows that the molybdenite is pure and has no mineral intergrowths. Molybdenite separates were obtained using a small hand-held drill and creating a molybdenite powder under a binocular microscope.

Re and Os isotopic concentrations were determined by isotope dilution using a Thermo Triton NTIMS (Negative Thermal Ionization Mass Spectrometry) machine at AIRIE. Precisely weighed samples were loaded into a Carius tube with 8 mL inverse aqua regia and Re-Os spikes for sample dissolution and sample-spike equilibration. A mixed Re-double Os spike (^185^Re-^188^Os-^190^Os) permits a mass fractionation correction for Os and assessment of any common Os in the molybdenite (Markey et al. 2003). Both samples had negligible common Os. All uncertainties are reported at 2-sigma (ESM Table 7) and include the ^187^Re decay constant uncertainty (λ). Re and Os blanks (Re blank = 11.77 ± 0.03 pg, Os blank = 0.130 ± 0.003 pg with ^187^Os/^188^Os = 0.350 ± 0.007) do not affect the calculated ages.

### Whole rock Rb–Sr and Sm–Nd radiogenic isotopes

Three whole rock samples, rich in actinolite, from the IOCG stage of the Abundancia vein were analyzed for their bulk rock Sr and Nd radiogenic isotope composition. Samples were initially crushed using a Plattner mortar-and-pestle and later reduced to powder in ethanol with an agate mortar-and-pestle. The samples might contain some minor contamination from the MtAp stage as the fine-grained nature of the veinlets makes it sometimes difficult to separate minerals from both events.

Rb–Sr and Sm–Nd bulk rock analyses were done at the Unidad de Geocronología (CAI de Ciencias de la Tierra y Arqueometría) of the Universidad Complutense de Madrid using Isotope Dilution-Thermal Ionization Mass Spectrometry (ID-TIMS) with an Isotopx Phoenix TIMS. The samples were spiked with ^84^Sr, ^87^Rb, and a mix of ^149^Sm-^150^Nd, and digested using ultra clean reagents. They were processed using chromatography, where Rb, Sr, and rare earth elements (REEs), were separated in DOWEX AG (50WÅ ~ 12 Resin, 200–400 mesh) columns. In order to isolate the Sm and Nd, REE fractions were separated in HDEHP-impregnated Teflon-powder columns. Fractionation effects were corrected using a normalization of ^86^Sr/^88^Sr = 0.1194 and ^146^Nd/^144^Nd = 0.7219. The procedural blank was 0.5 ng for Sr, and 0.1 ng for Nd. The standard materials used during the analyses were the following: Sr standard NBS-987 (0.710245 ± 0.000004; n = 58), Nd standards La Jolla (0.511850 ± 0.000004, n = 36), and JNdi-1 (0.512108 ± 0.000003, n = 33).

### *δ*^*34*^*S stable isotopes in sulfides*

The sulfur isotopic composition was determined in fourteen samples of chalcopyrite and pyrite from the IOCG event at the Abundancia vein. In four of them, two aliquots were taken for control. The sulfides were crushed in a stainless-steel mortar and handpicked under a binocular microscope using titanium tweezers, followed by magnetic separation to remove the magnetite. Pure sulfide grains were ground to ~1 micron size in an agate mortar using a pestle, obtaining a powder of ~0.5 g. Sulfides with other minerals attached to the surface were ground and sieved and retained to 63 microns, magnetite was removed with a magnet, and the sample was processed using heavy liquids, bromoform, and methylene iodide, in order to separate the sulfides from the other minerals.

The sulfur isotope measurements were done at the Stable Isotope Laboratory of the Instituto Andaluz de Ciencias de la Tierra (CSIC-UGR, Granada, Spain)*.* Samples were analyzed by combusting the samples with V_2_O_5_ and O_2_ at 1030 °C in a Carlo Elba NC1500 elemental analyzer online with a Delta Plus XL mass spectrometer (EA-IRMS). The stable isotope composition is reported as δ values per mil, calculated using the δ = (R_sample_/R_standard_ – 1)*1000 equation, where R = ^34^S/^32^S for δ^34^S. Commercial SO_2_ was used as the internal standard for sulfur isotopic analyses. For sulfur, five internal standards (organic and inorganic material) ranging between − 6.38 ‰ to + 23.25 ‰ (CDT, Canyon Diablo Troilite), along with the IAEA international reference materials IAEA-S1, IAEA-S2, IAEA-S3, NBS-127 and CP-1 were analyzed. This study used three internal standards of + 23.25 ‰, + 6.03, and − 6.38 ‰ (CDT). After correction of the mass spectrometer daily drift, the precision calculated from standards systematically interspersed in analytical batches was better than ± 0.2‰. The V-CDT (Vienna-Canyon Diablo Troilite) is the standard reference material for reporting sulfur isotope data.

## Results

### Mineral textures and chemical composition

#### Actinolite

Our study identifies two types of actinolite in the Montecristo system: 1) medium to coarse grained, pale to dark green actinolite I, related to the MtAp I and II events; and 2) fine grained dark green actinolite II, associated with the later IOCG stage. Both types can be distinguished based on mineral textures, associated mineral assemblage, and crosscutting relationships.

Early actinolite I in the Abundancia vein occurs as subhedral, coarse-grained (up to 0.5 cm long) crystals, often arranged in cm-thick bands hosted by, or interlayered with, the magnetite. Actinolite I also occur disseminated in the magnetite or enclosed by apatite crystals in a “poikilitic” type texture (see Fig. 4b and ESM Fig. 1) that is similar to those in cumulate rocks (Wager et al. 1960; McBirney and Noyes 1979), and chilled parts of mafic and ultramafic intrusions (Wager 1961). Later crosscutting magnetite-rich mineralization MtAp II event also contains actinolite I with the same characteristics but with a smaller (up to 200 µm) grain size. Actinolite I in the San Juan MtAp vein is intergrown with magnetite, coarse-grained (up to 7 mm) and forms massive aggregates, some moderately altered to chlorite. Actinolite appears to predate at least some of the magnetite (Fig. 4g).

Actinolite II is fine-grained (up to 200 µm) and occurs in veinlets paragenetically related to chalcopyrite, pyrite, quartz, and minor magnetite and titanite of the IOCG event. There are also abundant remnants, partially replaced, of actinolite I and apatite in the IOCG zone (see ESM Fig. 1).

**Fig. 5:**
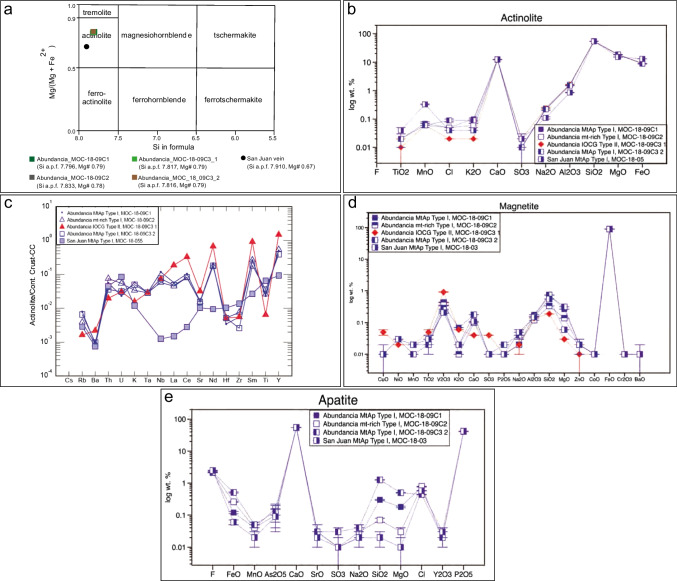
Chemical composition of minerals from the Abundancia and San Juan veins. For clarity, the graphs show the average compositions of analyses in the ESM. (a) Classification chart for calcic amphiboles (after Leake et al. 1997). (b) Plot comparing the composition of actinolite from the different events at Montecristo. Compositional plots showing the average trace element composition of: (c) actinolite; (d) magnetite; (e) fluorapatite

The chemical composition of the actinolite is in ESM Table 2B, and a comparative oxide composition is shown in Fig. 5b. All samples analyzed classify as actinolite (Fig. 5a; Leake et al. 1997), with the Mg# [molar (Mg/(Mg + Fe)] ranging from 0.67 to 0.79, and the Si a.p.f.u. between 7.796 and 7.910. In general, the actinolite samples show the following compositional range for major elements: MgO (15.20–18.70 wt.%), CaO (12.20–12.60 wt.%), FeO (8.70–13.10 wt.%), Al_2_O_3_ (0.90–1.70 wt.%), Na_2_O (0.10–0.20 wt.%), MnO (0.10–0.30 wt.%), TiO_2_ (0.01–0.04 wt.%), SO_3_ (0.01–0.02 wt.%), and small amounts of K_2_O (0.02–0.10 wt.%). It has between 190 and 940 ppm Cl and F is below the LOD.

The EPMA analyses show that actinolite I and II from Abundancia are relatively more depleted in Fe (range of 6.80–7.10 wt.%), while actinolite I from San Juan is enriched in Fe (average of 10.14 wt.%) and Mn (average of 2,562 ppm). Actinolite II in Abundancia has low average Ti (57 ppm), Cl (188 ppm), and K (197 ppm) compared to Actinolite I, which has an average of 112 ppm, 658 ppm, and 697 ppm, respectively. This is reinforced by the Fe, Ca, Mg, K, and Ti X-ray maps (ESM Fig. 3). Alternatively, the San Juan actinolite I is enriched throughout the grains in Ti (average of 253 ppm) compared to the other samples, but this can be due to the presence of microinclusions of a Ti–rich phase. In addition, the X-ray maps show the presence of abundant disseminated inclusions of titanite (< 10 µm) (ESM Fig. 3). The actinolite compositional data obtained in this study generally agree with other data previously published for actinolite in MtAp deposits in the Coastal Cordillera (Rojas et al. 2018) and IOCG (Del Real et al. 2021).

LA-ICPMS trace element data for actinolite are presented in the ESM Table 2B and Fig. 5c and are normalized to continental crust (Rudnick and Gao 2003). The data indicate that MtAp and IOCG-related actinolite, types I and II, respectively, from Abundancia generally have similar trace element patterns. Actinolite II has slightly higher Ba, Sr, and REE contents, and lower Rb, K, and Ti than actinolite I. Actinolite I from the San Juan vein, however, shows a different trace element composition. It is depleted in Nb, La, Ce, Nd, Sm, and Y and is slightly enriched in Hf, Zr, and Ti, suggesting different chemistry (i.e., heterogeneity) between individual veins.

### Magnetite

Magnetite from the MtAp stage in the Abundancia and San Juan veins have similar textures and are grouped as magnetite type I. In some cases, the magnetite grains exhibit exsolution textures, less than 100 µm in size, of titanite (ESM Fig. 1v). Magnetite I from the Abundancia vein hosts abundant acicular actinolite inclusions (Fig. 4b). At the same time, the San Juan magnetite is inclusion-free. Magnetite II from the IOCG event is relatively homogeneous and has only quartz inclusions (See ESM Fig. 1). Major, minor, and trace elements data for both types I and II of the magnetite samples are presented in ESM Table 2D and comparative oxide average composition graphs are plotted in Fig. 5d.

All the magnetite I samples have a nearly identical chemical composition and do not show noticeable systematic trends or generations. The most representative elements include very low Ti contents (100–190 ppm) as well as Cu (50–60 ppm), Ni (200–240 ppm), V (1,420–3,010 ppm), Cr (30–60 ppm), S (10–50 ppm), Si (1,570–3,510 ppm), Al (650–940 ppm), Mn (100–180 ppm), Mg (340–1,610 ppm), Ca (710–1,320 ppm), and Zn (130–160 ppm). The relatively constant contents suggest that these elements are not hosted in nanoinclusions as happens in other MtAp Coastal Cordillera deposits (e.g., Knipping et al. 2015b). Chemical compositions similar to magnetite I are reported in magnetite from other MtAp deposits in the CIB (e.g., Knipping et al. 2015b, Los Colorados; Salazar et al. 2019, Cerro Negro Norte).

Magnetite II has a quite similar composition but significantly higher Cu contents, which are constant throughout the crystals. The average composition of magnetite II includes: Ti (282 ppm), Cu (406 ppm), Ni (150 ppm), V (6,193 ppm), Cr (81 ppm), S (162 ppm), Si (871 ppm), Al (730 ppm), Mn (149 ppm), Mg (211 ppm), Ca (251 ppm), and Zn (106 ppm). The geochemistry of magnetite in IOCG deposits is variable; however, similarities exist with previously published data on IOCG deposits worldwide (Rusk et al. 2009; Zhang et al. 2017).

### Apatite

Apatite is abundant in the MtAp stage, but we have not found apatite in the IOCG stage. Apatite crystals in the Abundancia MtAp mineralization are generally euhedral, coarse-grained (up to 1 cm) and lack evidence in BSE or CL of any superimposed metasomatic alteration. The apatite commonly encloses small (< 250 µm) actinolite crystals in a “poikilitic-like” texture (Fig. 4b) and shows a yellow CL emission (caused by Mn^2+^) typical of apatite having a magmatic origin (Dempster et al. 2003; Bouzari et al. 2016) (see ESM Fig. 1). Apatite in the San Juan vein is also euhedral and coarse-grained (up to 2 cm) (Fig. 4h). The distribution of apatite is identical to that described in the nearby Carmen de Fierro and Fresia MtAp deposits (Tornos et al. 2021) and elsewhere in MtAp pegmatites.

The EPMA analyses of apatite are provided in ESM Table 2F, and the average chemical composition for each apatite sample is plotted in Fig. 5e. Our data are similar to published apatite composition data from other MtAp deposits in the Coastal Cordillera (Treloar and Colley 1996; La Cruz et al. 2019). All samples correspond to fluorapatite (2.10–2.50 wt.% F) with a minor chlorapatite component (0.40–0.80 wt.% Cl) and include Na (160–330 ppm), Mn (180–360 ppm), Mg (40–3,010 ppm), Fe (470–3,980 ppm), As (570–1,020 ppm), Sr (170–280 ppm), Y (140–200 ppm), Si (85–5,890 ppm), and S (50–110 ppm). However, the highly variable Fe, Si, and Mg contents are most likely due to the presence of micro- or nanoinclusions of actinolite that was not visible with BSE or EPMA.

### *Actinolite *^*40*^*Ar/*^*39*^*Ar dates*

**Fig. 6:**
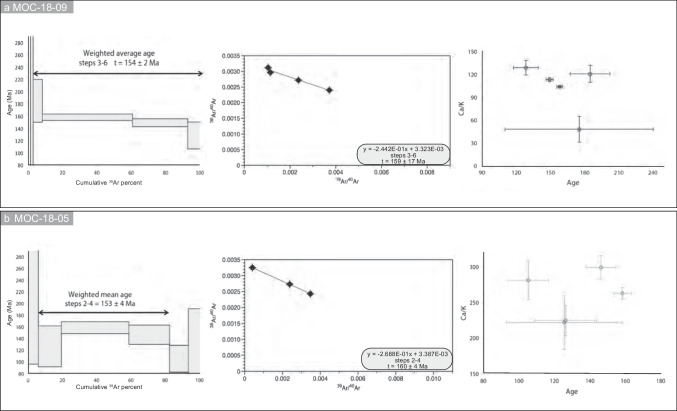
^40^Ar/.^39^Ar dating results. (a) Plateau age, isochron age, and Ca/K ratios for sample MOC-18–09. (b) Plateau age, isochron age, and Ca/K ratios for sample MOC-18–05

^40^Ar/^39^Ar data and plots for the two actinolite samples are reported in Fig. 6, and the full data set is available in the ESM Table 4. Actinolite MOC-18–09 from the Abundancia vein yielded a well-constrained plateau age of 154 ± 2 Ma (2-sigma), representing 92.3% of the ^39^Ar released. The plateau age is indistinguishable from the isochron age (159 ± 17 Ma, 2-sigma). The three plateau steps show a constant Ca/K ratio. In contrast, the Ar-poor low-temperature steps show lower ratios, indicating the presence of a contaminant phase with a higher amount of K or lower Ca content. The broadly constant Cl/K ratios mean that the sample is homogeneous and likely contains little if any, Cl-rich phase such as fluid inclusions. The coarse-grained actinolite MOC-18–05 from the San Juan vein yielded a plateau age of 153 ± 4 Ma (2-sigma), representing the 76.2% of the ^39^Ar released, and homogeneous Ca/K and Cl/K ratios in the plateau steps. The plateau age is indistinguishable at the 2-sigma level from the isochron age (160 ± 4 Ma).

A literature search of K–Ar dates of actinolite associated with the alteration halo in the Mantos deposit in the Montecristo district has an average age of 164 ± 11 Ma (JICA-MMAJ 1986), an age consistent at the 1-sigma level with the present results.

### Zircon U–Pb age and Hf tracer isotopes from the host rock

**Fig. 7:**
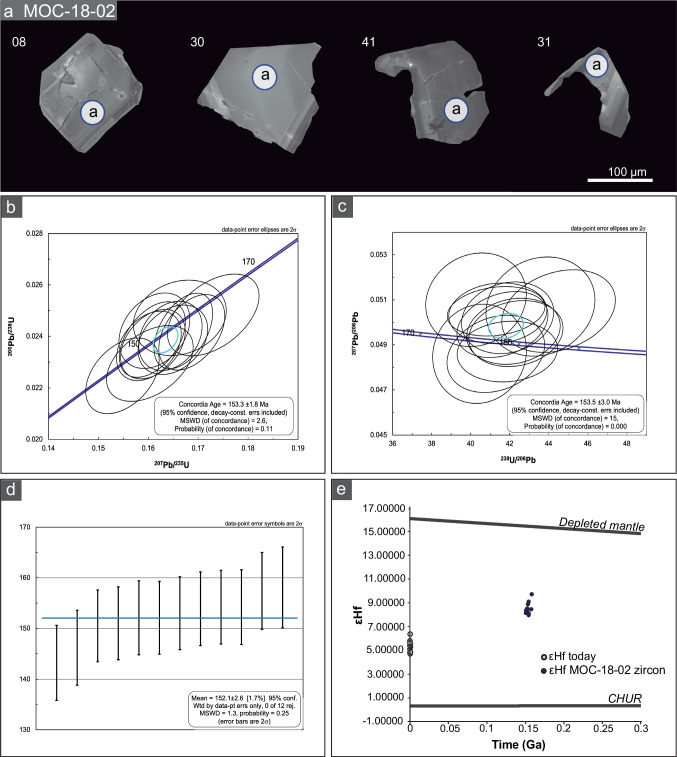
U–Pb and Hf zircon results from sample MOC-18–02. (a) CL images of representative zircon grains from the host diorite after the chemical abrasion treatment process. (b) Concordia diagram. (c) Tera-Wasserburg diagram. (d) Ordered weighted mean age plot. (e) εHf_i_ versus age plot

The U–Pb zircon data from the diorite host rock (sample MOC-18–02) are reported in the ESM Table 5 and in Fig. 7b to d. The Lu–Hf data are presented in Fig. 7e, and all the data and calculations are shown in the ESM Table 6. The zircon grains are of relatively small size (up to 100 μm long), euhedral to subhedral, and inclusion-free. Some grains show regular growth zoning, a characteristic of igneous zircon (Fig. 7a). No inherited cores were identified in any of the zircon grains.

The Concordia diagram shown in Fig. 7b yields a Concordia age of 153.3 ± 1.8 Ma (2-sigma; MSWD = 2.6; n = 12). This date overlaps with the weighted mean ^206^Pb/^238^U age of 152.1 ± 2.6 Ma (2-sigma; MSWD = 1.3; n = 12]), and with the inverse isochron (i.e., Tera-Wasserburg) date of 153.5 ± 3.0 Ma (2-sigma; MSWD = 15; n = 12) also obtained in this study. The Concordia age agrees with previously reported U–Pb dates for the earliest crystallization stages of the Matancilla Intrusive Complex (Álvarez et al. 2016; Mavor et al. 2022 and references therein). In situ initial Hf values from the above mentioned 12 individual zircon grains, and calculated using the Concordia age, range from εHf_i_ of + 8.0 (analysis 11a) to an εHf_i_ of + 9.7 (analysis 37a), which suggests a dominantly juvenile source, i.e., produced in the primitive mantle, but with some incorporation of less radiogenic Hf inherited from continental crust through assimilation during emplacement (εHf_i_ =  + 10.59 to + 16.56 for Mesozoic rocks of the Canadian Cordillera, Vervoort and Blichert-Toft 1999).

### Molybdenite Re-Os ages

The Re-Os ages for two molybdenite samples belonging to the IOCG stage are presented in ESM Table 7. The data show a significant age difference between the two samples: 162.4 ± 0.6 Ma (2-sigma) (MOC-18-07A) and 151.8 ± 0.6 Ma (2-sigma) (MOC-18-07G). Re concentrations are very different, with 07A yielding 23.14 ppm and 07G yielding 369.7 ppm Re. The geologically unrealistic old age for sample MOC-18-07A could reflect within-molybdenite mobilization and redistribution of Re and Os or incipient oxidation (Stein et al. 2003). In an earlier study of a Chilean IOCG deposit at Raúl-Condestable (De Haller et al. 2006), two Re-Os ages several million years older than the associated intrusion were explained by Re loss. At Raúl-Condestable, however, molybdenite is markedly early in the paragenesis, preceding chalcopyrite and pyrite. In our study, molybdenite is late in the paragenesis (Fig. 2), and follows chalcopyrite ore mineralization, making a direct analogy difficult. Given the association of our dated samples with a zone of intense slickenside, we suggest that redistribution of Re and radiogenic Os within molybdenite is the most plausible explanation.

### Sr and Nd radiogenic isotopes

**Fig. 8:**
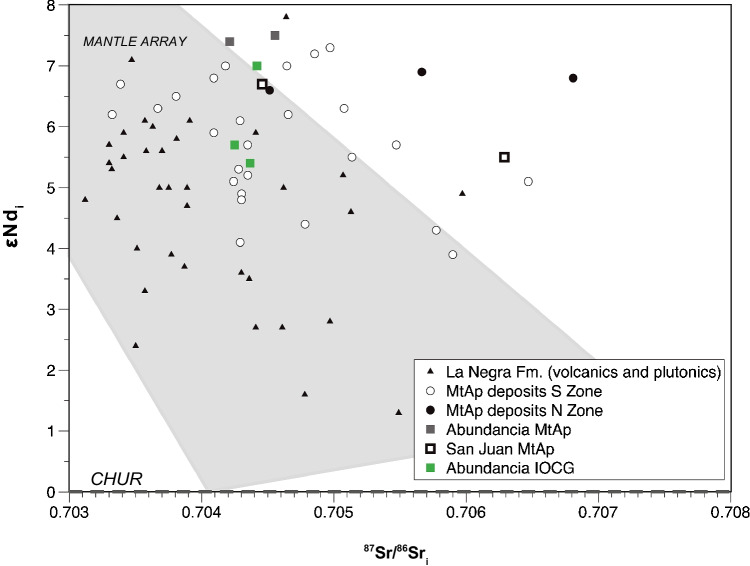
Initial Sr and Nd isotopic compositions of Cretaceous and Jurassic MtAp deposits in the Coastal Cordillera, including the Montecristo system and the host volcanic and plutonic rocks (data from Table 1 and Tornos et al. 2021)

Whole rock Rb–Sr and Sm–Nd results are shown in Fig. 8 and Table 1. The approximate deposit formation age of 154 Ma calculated by ^40^Ar/^39^Ar geochronology was used to calculate the initial ^87^Sr/^86^Sr and εNd_i_ values. These calculations yield εNd_i_ values of + 5.4 to + 7.0 and ^87^Sr/^86^Sr_i_ isotopic compositions of 0.70425 to 0.70442 for the IOCG event. This suggests a dominant juvenile source with values similar to the associated igneous rocks at the district scale. Other studies were previously done on calcite at Montecristo (^87^Sr/^86^Sr_i_ =  ~ 0.7058), Julia (^87^Sr/^86^Sr_i_ =  ~ 0.7046 to ~ 0.7048), and the Toldo-Velarde deposit in the Gatico district (^87^Sr/^86^Sr_i_ =  ~ 0.7041 to ~ 0.7043), suggesting similar ^87^Sr/^86^Sr_i_ values, and thus a similar source (Vivallo and Henríquez 1998).

**Table 1:** Whole-rock Rb–Sr and Sm–Nd data from the Abundancia IOCG mineralization

Sample	Description	Rb	Sr	^87^Sr/^86^Sr	StdErr*10^–6^ (2σ)	^87^Sr/^86^Sr_i_	Sm	Nd	^143^Nd/^144^Nd	StdErr*10^–6^ (2σ)	εNd_i_
MOC-18-06b	Magnetite-actinolite-sulfides	0.6	12.4	0.704556	2.8	0.7042492	2.17	14.9	0.512822	1.5	+ 5.7
MOC-18-09b	Magnetite-actinolite-sulfides	0.6	6.7	0.704935	3.8	0.7043673	0.54	5.2	0.512781	3.6	+ 5.4
MOC-18–12	Magnetite-actinolite-sulfides	0.5	5.0	0.705051	6.5	0.7044176	0.42	4.5	0.512858	1.9	+ 7.0

Notes: Rb, Sr, Sm, and Nd are ppm

The εNd_i_ value of + 7.0 is interpreted as inherited from the MtAp stage during the replacement of the MtAp by the IOCG mineralization. One MtAp value with a relatively low εNd_i_ value of + 5.1 and high ^87^Sr/^86^Sr_i_ ratios (0.70629) from Tornos et al. (2021) is also here interpreted as being partially re-equilibrated with the late IOCG-related fluids (see below).

### *δ*^*34*^*S isotopes in sulfides*

**Fig. 9:**
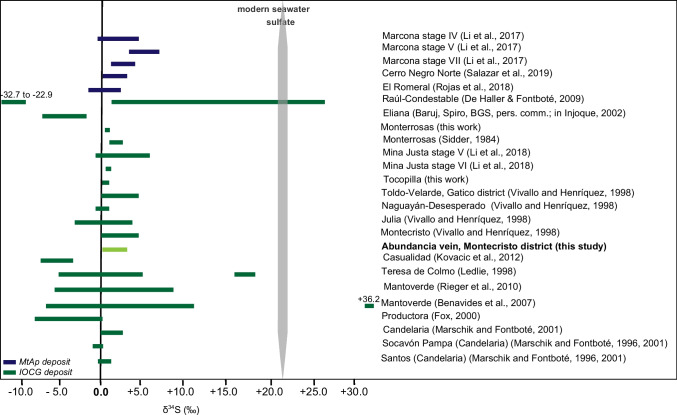
δ^34^S compositions for MtAp and IOCG deposits in Chile and Peru

δ^334^S values of sulfides from the IOCG event are listed in Table 2 and shown in Fig. 9. Sulfur isotopes of fifteen chalcopyrite and pyrite aliquots show a very restricted range between + 0.3 to + 3.4 per mil, with a mean of + 1.8 per mil. δ^34^S values in the Abundancia IOCG event agree with previously published sulfur isotopic studies in the Montecristo deposit (0 to + 5‰, Vivallo and Henríquez 1998). In addition, they are similar to other sulfur isotopic compositions determined for IOCG deposits in the Coastal Cordillera of Chile (Tocopilla, − 0.2 to + 0.6‰, unpub. data, F. Tornos; Gatico district, 0 to + 5‰, Vivallo and Henríquez 1998; Naguayán-Desesperado, − 1 to + 1‰, Vivallo and Henríquez 1998; Candelaria, 0.3 to + 3.1‰, Marschik and Fontboté 2001b; and Julia, − 4 to + 4‰, Vivallo and Henríquez 1998) or the sparse sulfides in the MtAp mineralization such as Los Colorados (− 3.2 to + 2‰; Tornos et al. 2021) (Fig. 9). These data suggest that the sulfur is dominantly of a juvenile magmatic derivation (δ^34^S = -3 to + 2‰; Ohmoto and Rye 1979; Ohmoto and Goldhaber 1997), with only a minor contribution of either crustally contaminated igneous rocks or a sedimentary source (Poulson et al. 1991). However, hematite-rich IOCG deposits show a wider range of δ^34^S values with a systematic displacement towards positive values, indicating a likely input of sulfur derived from the abiogenic reduction of seawater sulfate, or microbial reduction in a closed system but external to the magmatic-hydrothermal system, such as in Raul-Condestable (+ 1.0 to + 26.3‰ main ore stage; De Haller and Fontboté 2009), Teresa de Colmo (− 5.5 to 18.2‰; Ledlie 1998), and Mantoverde (− 6.8 to + 11.2 main ore stage, and + 26.4 to + 36.2 in later stages; Benavides et al. 2007; Rieger et al. 2010). This is consistent with previous studies by Chen (2013) suggesting the importance of external sulfur with δ^34^S values >  + 10‰ in the ore-forming processes of hematite-rich IOCG systems.

**Table 2:** δ^34^S results from the Abundancia IOCG mineralization

Sample name	Mineral	Sample description	δ^34^S ‰ (CDT)
MOC-18–6-1	ccp	Magnetite, sulfides disseminated and in veinlets (ccp, py), fine-grained actinolite, apatite (915 m)	1.9
MOC-18–6-2	ccp	Magnetite, sulfides disseminated and in veinlets (ccp, py), fine-grained actinolite, apatite (915 m)	2.0
MOC-18–9-1	ccp	Magnetite, apatite, fine-grained actinolite, sulfides, disseminated and in veinlets (py, ccp) (940 m)	0.6
MOC-18–9-2	py	Magnetite, apatite, fine-grained actinolite, sulfides, disseminated and in veinlets (py, ccp) (940 m)	0.3
MOC-18–11-a-1	ccp	Late massive chalcopyrite vein with quartz (940 m)	3.1
MOC-18–11-a-2	ccp	Late massive chalcopyrite vein with quartz (940 m)	3.1
MOC-18–11-b-1	ccp	Magnetite, sulfides disseminated and in veinlets (ccp, py) (940 m)	1.2
MOC-18–11-b-2	ccp	Magnetite, sulfides disseminated and in veinlets (ccp, py) (940 m)	1.2
ABU-3	py	Magnetite, sulfides disseminated and in veinlets (ccp, py), fine-grained actinolite, apatite (915 m)	3.4
ABU-4	py	Magnetite, sulfides disseminated and in veinlets (ccp, py), fine-grained actinolite, apatite (915 m)	1.8
ABU-5	py	Magnetite, sulfides disseminated and in veinlets (ccp, py), fine-grained actinolite, apatite (915 m)	0.5
MOC-18–12	ccp	Magnetite, fine-grained actinolite, and sulfides (ccp, py)	2.0
MOC-18–12	ccp	Magnetite, fine-grained actinolite, and sulfides (ccp, py)	1.6
MOC-18-06b	ccp	Magnetite, apatite, fine-grained actinolite, and sulfides (ccp, py)	2.0
MOC-18-06c	ccp	Magnetite, apatite, fine-grained actinolite, and sulfides (ccp, py)	2.4
*CDT* Canyon Diablo Troilite (CDT) standard. Analytical error better than ± 0.2 per mil

## Discussion

### Timing of mineralization

The U–Pb zircon crystallization age of 153.3 ± 1.8 Ma (2-sigma) for the host diorite is consistent within error with the ^40^Ar/^39^Ar dates of the MtAp event (153 ± 4 Ma and 154 ± 2 Ma, 2-sigma), and the Re-Os molybdenite formation age of 151.8 ± 0.6 Ma (2-sigma) for the IOCG mineralization. The dates obtained in the present study indicate that the crystallization of the host diorite, the formation of the MtAp rocks, and the hydrothermal IOCG mineralization, are coeval within error and took place in a period of ~ 3.4 Ma that cannot be temporally resolved with geochronology; probably, the mineralization formed soon after the crystallization of the host diorite, especially considering that the Ar cooling ages are younger than zircon dates (Renne et al. 1998; Min et al. 2000; Kuiper et al. 2008).

The age of the host diorite indicates that it is related to one of the youngest pulses of the Matancilla Igneous Complex and suggests that mineralization took place during the waning stages of magmatic activity.

### The evolution of the Montecristo system

The Montecristo vein system consists of multi-stage mineralization with an early MtAp stage (MtAp I and II) crosscut by a younger IOCG mineralization event (Events III and IV) (see Fig. 2).

The textures of the early MtAp stage are similar to those of the intrusive part of the El Laco deposit or the MtAp deposits of the Coastal Cordillera that include the diagnostic pegmatite-like textures with sometimes banded, coarse-grained fluorapatite with unidirectional growth textures intergrown with diopside or actinolite and magnetite (Naranjo et al. 2010; Tornos et al. 2017, 2021). In the case of the Abundancia vein, they also include “poikilitic” fluorapatite enclosing well-formed acicular, mostly euhedral actinolite crystals. Some of these rocks likely represent the magmatic to hydrothermal transition in MtAp systems and a pulsatile regime similar to granite-related pegmatite (Tornos et al. 2021).

The direct crystallization of immiscible melts can explain the formation of MtAp mineralization. Geological (Naslund et al. 2002; Chen et al. 2010a; Mungall et al. 2018), isotopic (Tornos et al. 2016, 2017, 2021; Troll et al. 2019; Weis et al 2021), experimental (Lledó 2005; Veksler et al. 2007; Hou et al. 2018; Mungall et al. 2018; Lledó et al. 2020, between many others), numerical modeling (Keller et al. 2022), and melt inclusion data (Jacobsen et al. 2005; Naslund et al. 2009; Velasco et al. 2016; Bain et al. 2020, 2021 Pietruszka et al. in 2022) provide overwhelming evidence, often times neglected by supporters of alternative models, that these rocks crystallized from iron-rich silica-poor immiscible melts.

Overall, the composition of magnetite and actinolite in the IOCG stage (Event III) is similar to that of the MtAp stage. The actinolite and magnetite EPMA X-ray maps and compositions (ESM Fig. 3) show only slight chemical differences, suggesting that magnetite and actinolite compositions are not useful tracers for discriminating types of mineralization, at least within these vein-like MtAp and IOCG systems. The only observed major difference is that magnetite II is distinctive in containing abundant quartz inclusions not present in magnetite I. Furthermore, magnetite II has, on average, an order of magnitude more Cu (avg. 406 ppm) than magnetite I (50–64 ppm), something consistent with the abundance of Cu during the IOCG event.

These results suggest that the Montecristo MtAp-IOCG system has a much simpler mineralogical evolution of magnetite and actinolite than that described by, for example, Knipping et al. (2015b), Rojas et al. (2018), Huang and Beaudoin (2019), Palma et al. (2020) in other MtAp and IOCG deposits of the Coastal Cordillera. Our preferred interpretation is that the monotonous compositions at Montecristo are due to the fact that the mineralization is paragenetically simple and infills structures with little, if any, interaction with the host rocks.

For example, the Los Colorados MtAp system has very different types of ore, including massive and pegmatite infilling structures, as well as large amounts of disseminated and stockwork-like magnetite ore replacing andesite (see Knipping et al 2015b; Tornos et al 2021). The stratabound IOCG deposits of the CIB also include very different types of magnetite from the replacement of different types of andesitic rocks or direct precipitation along structures (del Real et al. 2021). Magnetite, in both cases, can have abundant micro- and nanoinclusions and/or records dramatic changes in fluid compositions, *f*O_2_ and temperatures, all influencing to an unknown extent in an unbuffered system the chemical composition of the minerals.

### Different sources for the IOCG and MtAp mineralization

Except for one sample by Tornos et al. (2021), the Montecristo mineralization has rather restricted ^87^Sr/^86^Sr_i_ values (0.7042–0.7047). However, variable εNd_i_ compositions (+ 5.4 to + 7.5) that plot in the mantle array of Fig. 8 are within the field of Late Jurassic-Early Cretaceous igneous rocks of the Coastal Cordillera. Specifically, the IOCG mineralization has lower (+ 5.4 to + 7.0), more crustal values than the MtAp rocks (+ 6.7 to + 7.5). The ultimate reason why sample ABU-7 of Tornos et al. (2021) has such a high radiogenic ^87^Sr/^86^Sr_i_ value is unknown but could reflect some inheritance from an old crustal basement or Sr-enriched slab fluids. Extremely radiogenic Sr values are a constant in some MtAp deposits of the CIB.

Nd isotopes are more robust and more difficult to reset than Sr isotopes and are a more reliable tracer of the source of fluids. Initial εNd values of the MtAp mineralization in Montecristo at 154 Ma are similar to those of nearby MtAp rocks, such as the Julia and Tocopilla vein deposits, and also of Jurassic age, that are + 7.5 and + 6.6. to + 6.8, respectively (Tornos et al. 2021). The lower εNd_i_ values of the IOCG mineralization cannot solely be derived from the same source as the MtAp mineralization or fluids in equilibrium. The εNd_i_ values of the Cu-Au mineralization probably inherited some Nd from the replaced MtAp rocks mixed with Nd derived from a reservoir with a significantly larger contribution of continental crust.

The εNd_i_ values for the host diorite calculated from the εHf_i_ of the zircon following Vervoot et al. (2011), assuming that there is no inheritance and that the zircon Hf is representative of the bulk rock, are between + 4.3 and + 5.4 (see ESM Table 6). Thus, our best interpretation is that the εNd_i_ values of the IOCG likely track mixing between the replaced MtAp mineralization and the Nd inherited from the host diorite or a non-exposed underlying intrusion of equivalent isotopic composition. The relatively low εNd_i_ values of the IOCG mineralization at Montecristo are broadly similar to those of the MtAp deposits located between Taltal and La Serena (Chile), as well as to the εNd_i_ values of several intrusions in the Coastal Batholith (Lucassen et al. 2006). In particular, the εNd_i_ values are like those of the Copiapó Plutonic Complex (CPC) of Early Cretaceous age (+ 4.7 to + 5.8; Marschik et al. 2003) that is thought to be the source of the ore forming fluids of the IOCG mineralization in the Punta del Cobre district deposits (Marschik and Fontboté 2001b; Arévalo et al. 2006).

The sulfur isotope results in this study from both Events III and IV in the IOCG mineralization are consistent with the sulfur being derived from an H_2_S-bearing magmatic-hydrothermal fluid that transported the Cu, Mo, and Au (i.e., Rusk et al. 2004; Audétat et al. 2008, 2019; Kamenetsky and Kamenetsky 2010); derivation of reduced sulfur from the hydrothermal leaching of juvenile igneous rocks seems unlikely. These fluids also likely precipitated magnetite II, actinolite II, quartz and sulfides by cooling and interacting fluids with the older MtAp mineralized rocks.

Thus, the MtAp rocks only acted as structural and geochemical traps for the later IOCG mineralizing fluids due to the brittle and oxidized nature of the magnetite in a scheme similar to that proposed by Bauer et al (2018) in the Malmberget-Kiruna area. Such fluid-rock interactions would also create the calcic-iron-alkali alteration observed in the deeper parts of the Montecristo system.

**Fig. 10:**
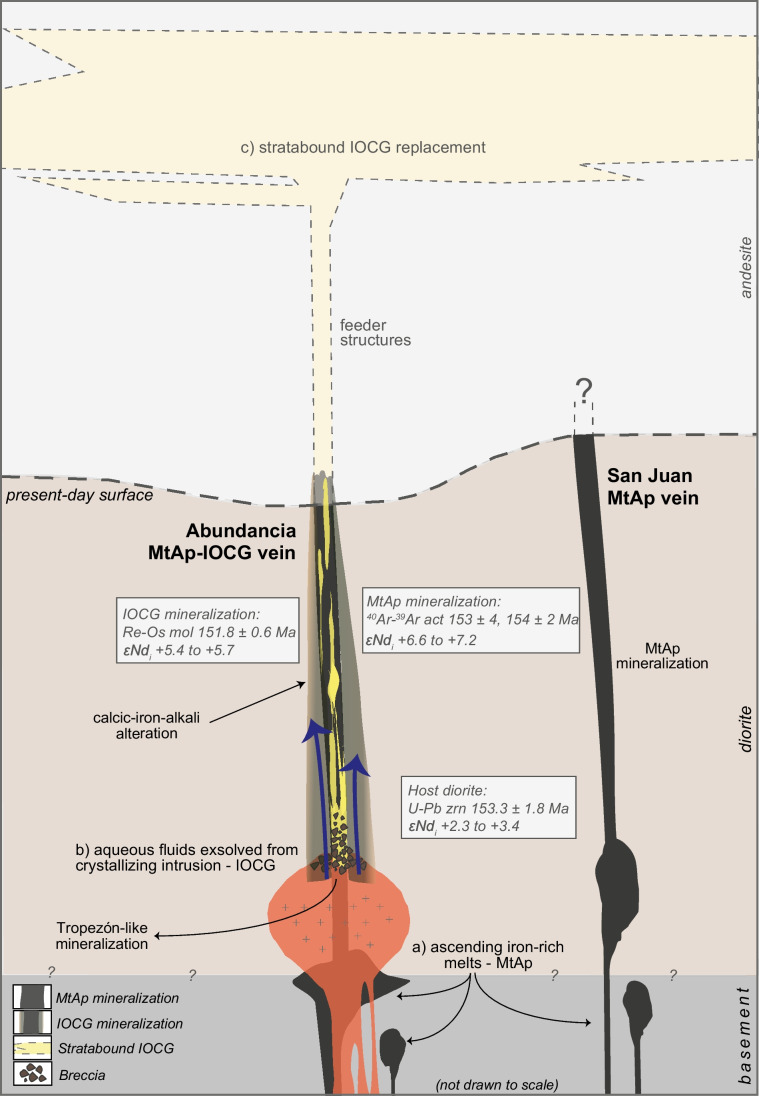
A genetic model for the formation of the MtAp-IOCG system in the Montecristo district. (a) Ascent of iron-rich melts after separating from a silicate melt with later crystallization along tensional faults related to the Atacama Fault System. The intrusion of these dykes postdates the emplacement and crystallization of the Matancilla Intrusive Complex. (b) A late magmatic-hydrothermal event with replacement of the earlier MtAp rocks by the IOCG mineralization. Fluids are derived from a deep, late crystallizing, diorite intrusion. (c) Local replacement of Late Jurassic-Early Cretaceous andesite by the IOCG mineralization, forming stratabound deposits. Not to scale

In the Coastal Cordillera, the formation of iron-rich melts during the Middle-Late Jurassic seems to be controlled by the contamination of the mantle wedge by Sr-rich crustally derived dehydration fluids. This would promote partial melting of the mantle wedge, triggering the separation of the iron-rich melts in the most contaminated zones from a parental mafic melt (Tornos et al. 2021). After the emplacement and crystallization of the MIC, these iron-rich melts ascended along restricted extensional domains within the transcrustal faults of the AFS and when attaining neutral buoyancy crystallized magnetite I, actinolite I, and fluorapatite at ca. 800–1200° C (Fig. 10; Bain et al. 2021 and references therein) coeval with dewatering. High temperatures and the oxidized nature of these iron-rich melts—systematically enriched in anhydrite (Tornos et al. 2017, 2021)—and probably the low Cu contents manifested in the magnetite composition inhibit the formation of significant amounts of sulfides. The small amounts of available reduced sulfur allowed the precipitation at temperatures below ca. 700 °C of the sparse amounts of sulfides found in MtAp systems. The homogeneous structures in the MtAp veins and the lack of explosion breccias suggest that these veins crystallized below the two-phase surface at ca. 0.2 GPa fluid pressure.

Geologic features and Lu–Hf, Sr–Nd, and S isotope compositions are consistent with the source of the IOCG mineralization event at Montecristo being magmatic-hydrothermal fluids derived from more crustal, but likely, primitive intrusions of likely dioritic composition. Here, upwelling fluids derived from the crystallization of the diorite would react with the earlier MtAp mineralization, promoting the formation of large amounts of magnetite II, actinolite II, quartz, and sulfides. Chalcopyrite and pyrite would precipitate due to the alkalinization and oxidation of fluids due to a reaction with magnetite I, a classical mechanism for destabilizing the chloride complexes that control the transport of Cu at high temperatures (Liu and McPhail 2005). A drop in the aH_2_S_aq_ due to sulfide precipitation would, in turn, destabilize the HS complexes that control gold solubility (Shenberger and Barnes 1989; Loucks and Mavrogenes 1999; Zezin et al. 2007; Pokrovski et al. 2014), leading to its precipitation. Later collapse of the hydrothermal system should lead to the formation of the late calcite veins. Again, we have not found any evidence suggesting that the IOCG mineralization precipitated above the two-phase surface; thus, these veins are also probably deep.

In this scenario, the brecciated monzogabbro found underneath the Abundancia vein and recorded by Díaz et al. (2018) may represent the upper part of a crystallizing subvolcanic cupola that would be the deep magmatic-hydrothermal root of the IOCG vein-like mineralization. Equivalent breccias related to Cu-Mo-(Au) mineralization hosted by diorite have previously been described by Tornos et al. (2010) a few km from the Montecristo district in the Tropezón IOCG deposit. There, breccia pipes supported by tourmaline or quartz grade into a replacive IOCG-like mineralization with magnetite, sulfides, and actinolite, replacing mafic plutonic rocks. In addition, a direct link between the IOCG mineralization and the nearby silicate intrusions has previously been proposed for other IOCG deposits in the Coastal Cordillera (Boric et al. 1990; Vila et al. 1996; Hopper and Correa 2000; Marschik and Fontboté 2001b; Ray and Dick 2002; Gelcich et al. 2003; Sillitoe 2003; Tornos 2011).

Erosion prevents our knowledge of the mineralization that may have originally overlain these vein-like deposits. However, despite the age difference, these veins likely represent the roots of the abundant stratabound IOCG deposits in the Coastal Cordillera, such as in the Punta del Cobre district. The mineralization replaces favorable horizons in the Late Jurassic-Early Cretaceous andesite (Marschik and Fontboté 2001b). However, it is rooted in large NNW-SSE to WNW-ESE tensional sub-vertical structures infilled with an assemblage similar to the IOCG stage at Montecristo. The late calcite veins are identical to the uppermost part of the feeder structures in the Punta del Cobre district (N. Pop, pers. com., 2015).

The data obtained in this research might lead to different interpretations. However, based on our petrographic, geochemical, mineralogical, geochronological stable and radiogenic isotope, and field data, these data suggest that these systems reveal the superposition of genetically unrelated MtAp and IOCG systems that were channelized along deep-rooted tensional structures. The existence of independent sources for MtAp and IOCG systems most likely explains why, at a global scale, they are only rarely juxtaposed. Their similar mineralogy also can guide the misidentification of these mineralized systems in other locations. However, the large size of some of these IOCG deposits is likely due to the fact that magnetite is an excellent geochemical trap, but the copper-rich event can be several million years younger than the associated magnetite-rich rock (Rotherham 1997; Bauer et al. 2018).

## Conclusions

Montecristo is one of the few places worldwide with a clear superposition of an IOCG mineralization on an older MtAp system. The Montecristo vein system is hosted by a very late pulse of diorite in the late Jurassic Matancilla Intrusive Complex. It includes the early formation at ca. 154 Ma of a MtAp assemblage, including magnetite, fluorapatite, and actinolite, having relatively high εNd_i_ values. These rocks crystallized from iron-rich melts intruding along secondary structures of the transcrustal Atacama Fault System. This early magmatic event was most likely later followed by the circulation of magmatic-hydrothermal fluids along the same secondary structures, and the partial replacement of the earlier mineralization by an IOCG assemblage that precipitated from magmatic-hydrothermal fluids derived from the crystallization and dewatering of igneous rock with more crustal contamination, and likely equivalent to the host diorite. All mineralization events took place in a relatively short period of time, less than ~ 3.4 million years.

Although the genetic relationship between MtAp and IOCG deposits remains debatable, the petrographic, geochemical, mineralogical, geochronological stable and radiogenic isotope, and field data results from the present study suggest that the IOCG and MtAp mineralization events in the Montecristo district are genetically independent of each other.

## Supplementary Information

Below is the link to the electronic supplementary material.

Supplementary file1 (PDF 1032 KB)

Supplementary file2 (XLSX 33 KB)

Supplementary file3 (PDF 6161 KB)

Supplementary file4 (PDF 114 KB)

Supplementary file5 (PDF 148 KB)

Supplementary file6 (XLSX 19.7 KB)

Supplementary file7 (PDF 83 KB)

## Data Availability

All data are available in the Supplementary Materials.
