# Supplementary material for: The Montecristo mining district, northern Chile: the relationship between vein-like magnetite-(apatite) and iron oxide-copper–gold deposits
Source: Miner Depos. 2023 Mar 28;58(6):1023–49. doi: 10.1007/s00126-023-01172-0 (PMC10329088; doi:10.1007/s00126-023-01172-0)
Supplement: Supplementary file 1 — Supplementary file1 (PDF 1032 KB) [file 126_2023_1172_MOESM1_ESM.pdf]

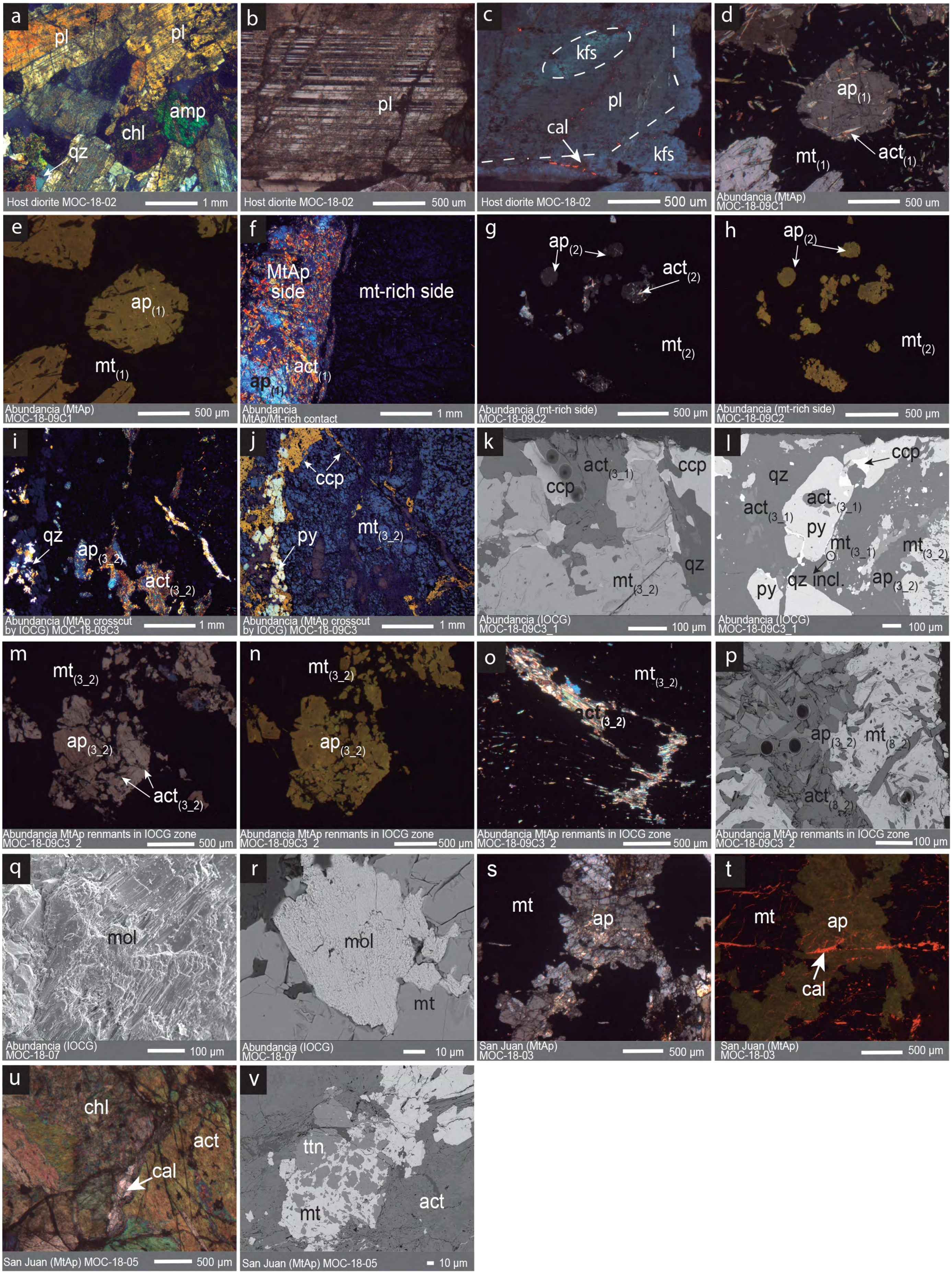
a
pl
pl
amp
chl
qz
Host diorite MOC-18-02
1 mm
b
pl
Host diorite MOC-18-02
500 um
c
kfs
pl
cal
kfs
Host diorite MOC-18-02
500 um
d
ap(1)
act(1)
mt(1)
Abundancia (MtAp) MOC-18-09C1
500 um
e
ap(1)
mt(1)
Abundancia (MtAp) MOC-18-09C1
500 μm
f
MtAp side
mt-rich side
act(1)
ap(1)
Abundancia MtAp/Mt-rich contact
1 mm
g
ap(2)
act(2)
mt(2)
Abundancia (mt-rich side) MOC-18-09C2
500 μm
h
ap(2)
mt(2)
Abundancia (mt-rich side) MOC-18-09C2
500 μm
i
qz
ap(3_2)
act(3_2)
Abundancia (MtAp crosscut by IOCG) MOC-18-09C3
1 mm
j
ccp
py
mt(3_2)
Abundancia (MtAp crosscut by IOCG) MOC-18-09C3
1 mm
k
act(3_1)
ccp
ccp
qz
mt(3_2)
Abundancia (IOCG) MOC-18-09C3_1
100 μm
l
ccp
qz
act(3_1)
act(3_1)
py
mt(3_1)
mt(3_2)
qz incl.
ap(3_2)
py
Abundancia (IOCG) MOC-18-09C3_1
100 μm
m
mt(3_2)
ap(3_2)
act(3_2)
Abundancia MtAp renmants in IOCG zone MOC-18-09C3_2
500 μm
n
mt(3_2)
ap(3_2)
Abundancia MtAp renmants in IOCG zone MOC-18-09C3_2
500 μm
o
mt(3_2)
act(3_2)
Abundancia MtAp renmants in IOCG zone MOC-18-09C3_2
500 μm
p
ap(3_2)
mt(8_2)
act(8_2)
Abundancia MtAp renmants in IOCG zone MOC-18-09C3_2
100 μm
q
mol
Abundancia (IOCG) MOC-18-07
100 μm
r
mol
mt
Abundancia (IOCG) MOC-18-07
10 μm
s
mt
ap
San Juan (MtAp) MOC-18-03
500 μm
t
mt
ap
cal
San Juan (MtAp) MOC-18-03
500 μm
u
chl
act
cal
San Juan (MtAp) MOC-18-05
500 μm
v
ttn
mt
act
San Juan (MtAp) MOC-18-05
10 μm

ESM Figure 1 Photomicrographs of the Abundancia and San Juan veins. **a** Dioritic host rock, XPL. **b** Plagioclase crystal in the diorite, XPL. **c** CL image of the plagioclase in the diorite being replaced by potassic feldspar and late calcite veinlets. **d** MtAp mineralization in Abundancia, with poikilitic euhedral apatite and acicular fine-grained actinolite crystals, also disseminated in the surrounding magnetite, XPL. **e** CL image of the previous sample showing the yellowish colour of the magmatic apatite. **f** Contact between the MtAp and the mt-rich side in the Abundancia vein, the mt-rich event being posterior, XPL. **g** Mt-rich side mineralization with poikilitic apatite and acicular fine-grained actinolite, surrounded by magnetite, XPL. **h** CL image of the previous sample showing magmatic apatite based on its colour. **i, j** IOCG event in Abundancia, with MtAp mineralization being crosscut by the ccp-py-qz-act-mt veinlets, XPL. **k, l** BSE images of the ccp-py-qz-act-mt IOCG veinlets. **m** Remnants of the MtAp mineralization in the IOCG zone, with coarse-grained poikilitic apatite enclosing acicular actinolite crystals. **n** CL image of the previous apatite. **o** Bands of acicular fine-grained actinolite in the magnetite from the MtAp mineralization in Abundancia. **p** BSE image of remnants of MtAp mineralization in the IOCG zone. **q** Secondary electron image of the laminar molybdenite, along with minor calcite. **r** BSE image of molybdenite crystals encrusted in the open spaces of the magnetite. **s** Coarse-grained apatite and massive magnetite in the San Juan vein, XPL. **t** CL image showing magmatic apatite, and late calcite veinlets in the San Juan vein. **u** Coarse-grained actinolite with weak to moderate chloritization, and late calcite veinlets, San Juan vein, XPL. **v** BSE images showing disseminated crystals in the actinolite with exsolution textures between magnetite and titanite in the San Juan vein. act=actinolite, amp=amphibole, ap=apatite, cal: calcite; chl: chlorite; ccp: chalcopyrite; kfs: potassic feldspar; mol: molybdenite; mt: magnetite; pl: plagioclase; qz: quartz; ttn: titanite
