# Supplementary material for: The Montecristo mining district, northern Chile: the relationship between vein-like magnetite-(apatite) and iron oxide-copper–gold deposits
Source: Miner Depos. 2023 Mar 28;58(6):1023–49. doi: 10.1007/s00126-023-01172-0 (PMC10329088; doi:10.1007/s00126-023-01172-0)
Supplement: Supplementary file 3 — Supplementary file3 (PDF 6161 KB) [file 126_2023_1172_MOESM3_ESM.pdf]

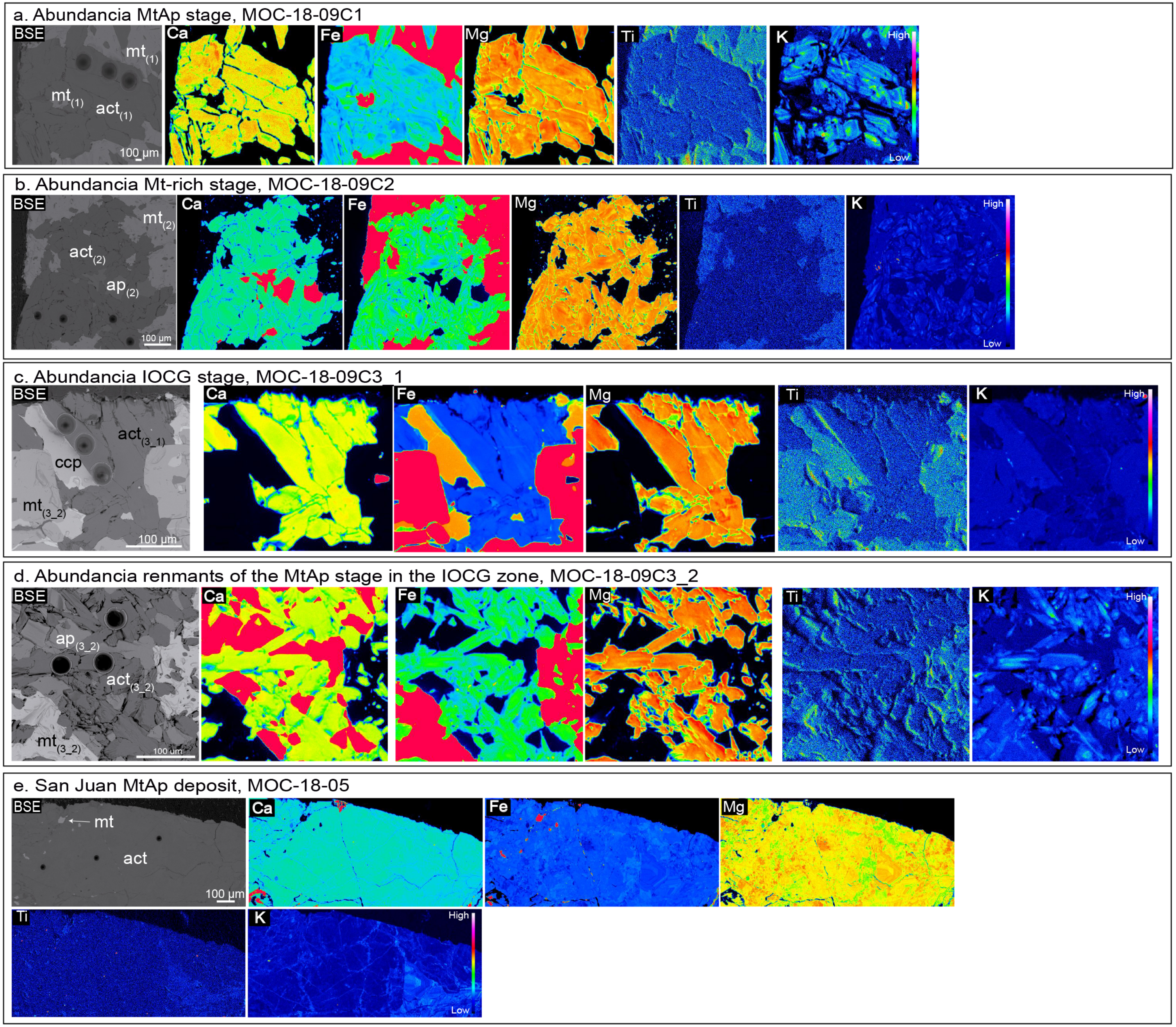

ESM Figure 3 EPMA X-ray maps of actinolite from samples in the Abundancia and San Juan deposits. **a** Sample MOC-18-09C1. **b** Sample MOC-18-09C2. **c** Sample MOC-18-09C3_1. **d** Sample MOC-18-09C3_2. **e** Sample MOC-18-05
