# Supplementary material for: The Montecristo mining district, northern Chile: the relationship between vein-like magnetite-(apatite) and iron oxide-copper–gold deposits
Source: Miner Depos. 2023 Mar 28;58(6):1023–49. doi: 10.1007/s00126-023-01172-0 (PMC10329088; doi:10.1007/s00126-023-01172-0)
Supplement: Supplementary file 4 — Supplementary file4 (PDF 114 KB) [file 126_2023_1172_MOESM4_ESM.pdf]

ESM Table 4. ^{40}Ar-^{39}Ar results.

^{40}Ar Total	Err.^{40}Ar	^{40}Ar*	Err.^{40}Ar *	^{39}Ar	Err.^{39}Ar	%^{39}Ar	^{38}Ar	Err.^{38}Ar	^{38}ArCl	Err.^{38}Cl	^{37}Ar	Err.^{37}Ar
**MOC 18-05**												
**Concentrations (g/g)**	**K=**	**3.19 E-04**	**Cl=**	**2.86 E-04**	**Ca=**	**1.02 E-01**						
**wt (g)**	**0.01467**											
**J**	**0.0011642**											
3.745 E-08	8.51E-12	4.005 E-10	1.11 E-10	3.219 E-12	2.66E-14	7.61	3.252 E-11	5.47 E-14	8.802 E-12	8.66 E-14	1.088 E-09	2.52E-12
1.166 E-08	2.10E-12	3.409 E-10	3.44 E-11	5.195 E-12	1.97E-14	12.28	2.600 E-11	4.35 E-14	1.863 E-11	5.53 E-14	6.335 E-10	1.53E-12
4.535 E-09	1.01E-12	1.248 E-09	1.11 E-11	1.720 E-11	2.08E-14	40.64	9.293 E-11	1.47 E-13	9.011 E-11	4.86 E-14	2.143 E-09	4.86E-12
3.722 E-09	8.85E-13	6.877 E-10	9.90 E-12	9.838 E-12	1.69E-14	23.25	4.942 E-11	7.82 E-14	4.702 E-11	4.85 E-14	1.460 E-09	3.34E-12
3.725 E-09	7.94E-13	2.274 E-10	1.12 E-11	4.692 E-12	1.27E-14	11.09	2.103 E-11	3.60 E-14	1.861 E-11	5.25 E-14	6.372 E-10	1.54E-12
1.073 E-08	2.46E-12	1.649 E-10	3.20 E-11	2.174 E-12	1.34E-14	5.14	1.517 E-11	2.79 E-14	8.397 E-12	5.95 E-14	3.040 E-10	8.64E-13

^{36}Ar	Err.^{36}Ar	Age	1 sigma Err Age	Age +2sigma	Age - 2sigma	Ca/K	Error Ca/K	Cl/K	Error Cl/K	39/40	error 39/40	36/40	error 36/40
1.244 E-10	3.71E-13	309.65	85.90	481.44	137.86	847.789	9.992	0.48667	0.00739	6.650 E-05	7.687 E-07	3.314 E-03	9.929 E-06
3.806 E-11	1.15E-13	144.15	14.57	173.30	115.01	257.614	1.279	0.63841	0.00336	4.093 E-04	1.779 E-06	3.251 E-03	9.893 E-06
1.156 E-11	3.59E-14	159.07	1.44	161.95	156.19	263.755	0.783	0.93266	0.00185	3.475 E-03	6.692 E-06	2.428 E-03	8.150 E-06
1.053 E-11	3.25E-14	156.08	2.28	160.65	151.52	319.793	1.093	0.85079	0.00233	2.380 E-03	6.067 E-06	2.731 E-03	8.900 E-06
1.188 E-11	3.74E-14	108.63	5.37	119.38	97.88	289.834	1.198	0.70583	0.00309	1.145 E-03	3.848 E-06	3.145 E-03	1.009 E-05
3.545 E-11	1.07E-13	167.74	32.54	232.83	102.65	299.330	2.252	0.68764	0.00683	1.837 E-04	1.280 E-06	3.298 E-03	9.982 E-06

(continued)

^{40}Ar Total	Err.^{40}Ar	$^{40}Ar^*$	Err.$^{40}Ar^*$	^{39}Ar	Err.^{39}Ar	%^{39}Ar	^{38}Ar	Err.^{38}Ar	$^{38}ArCl$	Err.^{38}Cl	^{37}Ar	Err.^{37}Ar
**MOC 18-09**												
**Concentrations (g/g)**	**K=**	**5.57 E-04**	**Cl=**	**4.31 E-04**	**Ca=**	**6.17 E-02**						
**wt (g)**	**0.01794**											
**J**	**0.0011644**											
2.552 E-08	5.80E-12	3.904 E-10	7.53 E-11	1.426 E-12	1.72E-14	1.69	2.115 E-11	3.76 E-14	5.253 E-12	7.01 E-14	3.691 E-11	3.78E-13
4.006 E-09	6.13E-13	1.138 E-10	1.21 E-11	7.948 E-13	1.20E-14	0.94	4.890 E-12	1.22 E-14	2.415 E-12	7.28 E-14	3.203 E-11	3.75E-13
3.610 E-09	8.44E-13	4.126 E-10	1.03 E-11	4.298 E-12	1.27E-14	5.09	2.612 E-11	4.55 E-14	2.398 E-11	5.09 E-14	2.771 E-10	7.53E-13
1.836 E-08	4.64E-12	3.457 E-09	4.55 E-11	4.501 E-11	4.47E-14	53.32	2.000 E-10	3.16 E-13	1.895 E-10	5.40 E-14	2.352 E-09	5.28E-12
6.985 E-09	1.45E-12	1.981 E-09	1.61 E-11	2.703 E-11	2.98E-14	32.01	1.106 E-10	1.72 E-13	1.068 E-10	4.63 E-14	1.561 E-09	3.53E-12
5.381 E-09	1.28E-12	3.706 E-10	1.56 E-11	5.868 E-12	1.43E-14	6.95	2.886 E-11	4.88 E-14	2.553 E-11	5.00 E-14	3.882 E-10	9.61E-13

^{36}Ar	Err.^{36}Ar	Age	1 sigma Err Age	Age +2sigma	Age - 2sigma	Ca/K	Error Ca/K	Cl/K	Error Cl/K	39/40	error 39/40	36/40	error 36/40
8.419 E-11	2.51E-13	506.97	97.98	702.93	311.01	51.105	0.817	0.65580	0.01188	5.489 E-05	6.729 E-07	3.298 E-03	9.881 E-06
1.304 E-11	4.06E-14	285.22	30.77	346.77	223.68	80.350	1.566	0.54096	0.01836	1.930 E-04	3.008 E-06	3.254 E-03	1.015 E-05
1.078 E-11	3.43E-14	199.34	5.01	209.36	189.31	130.721	0.546	0.99324	0.00379	1.139 E-03	3.624 E-06	2.967 E-03	9.539 E-06
5.050 E-11	1.51E-13	159.88	2.11	164.10	155.66	105.043	0.266	0.74934	0.00090	2.366 E-03	2.824 E-06	2.719 E-03	8.283 E-06
1.716 E-11	5.34E-14	153.43	1.26	155.96	150.90	116.523	0.304	0.70308	0.00096	3.719 E-03	4.897 E-06	2.400 E-03	7.711 E-06
1.688 E-11	5.21E-14	133.75	5.65	145.04	122.46	134.297	0.486	0.77450	0.00255	1.042 E-03	2.764 E-06	3.119 E-03	9.715 E-06
