# Supplementary material for: The Montecristo mining district, northern Chile: the relationship between vein-like magnetite-(apatite) and iron oxide-copper–gold deposits
Source: Miner Depos. 2023 Mar 28;58(6):1023–49. doi: 10.1007/s00126-023-01172-0 (PMC10329088; doi:10.1007/s00126-023-01172-0)
Supplement: Supplementary file 5 — Supplementary file5 (PDF 148 KB) [file 126_2023_1172_MOESM5_ESM.pdf]

ESM Table 5. Geochronology results for zircon from the host diorite (MOC-18-02): U-Pb-Th LA-ICPMS analytical data. Zircon grains with radiation damage in stages 2 and 3 were ignored for the final age calculations.

					Isotopic ratios											
Sample MOC-18-02 spot #	U approx. [ppm]	Th approx. [ppm]	U/th		$^{207}Pb/^{235}U$	2SE	$^{206}Pb/^{238}U$	2SE	rho	$^{238}U/^{206}Pb$	2SE	$^{207}Pb/^{206}Pb$	2SE	rho	$^{208}Pb/^{232}Th$	2SE
~~01a~~	~~3080~~	~~5400~~	~~0.938~~		~~0.1533~~	~~0.009~~	~~0.02297~~	~~0.0014~~	~~0.43549~~	~~43.53505~~	~~2.65342~~	~~0.0496~~	~~0.0024~~	~~0.33989~~	~~0.00592~~	~~0.00053~~
~~03a~~	~~2200~~	~~4630~~	~~0.848~~		~~0.1615~~	~~0.0064~~	~~0.0234~~	~~0.0012~~	~~0.39051~~	~~42.73504~~	~~2.191541~~	~~0.05~~	~~0.0016~~	~~0.16524~~	~~0.00641~~	~~0.00047~~
~~06a~~	~~5720~~	~~26300~~	~~0.4904~~		~~0.1582~~	~~0.0042~~	~~0.02332~~	~~0.0011~~	~~0.53561~~	~~42.88165~~	~~2.022719~~	~~0.04931~~	~~0.00063~~	~~0.22631~~	~~0.00643~~	~~0.00042~~
~~07a~~	~~3470~~	~~11060~~	~~0.836~~		~~0.1592~~	~~0.0049~~	~~0.02331~~	~~0.0011~~	~~0.27556~~	~~42.90004~~	~~2.024455~~	~~0.0496~~	~~0.0011~~	~~0.34411~~	~~0.00646~~	~~0.00044~~
08a	1484	6090	0.801		0.1601	0.0053	0.02363	0.0011	0.35949	42.31909	1.969996	0.0495	0.0012	0.16461	0.00634	0.00044
11a	2583	1060	4.66		0.167	0.0048	0.02421	0.0012	0.46667	41.30525	2.047348	0.05049	0.0009	0.19352	0.00667	0.0005
~~12a~~	~~3380~~	~~19700~~	~~0.412~~		~~0.1459~~	~~0.0045~~	~~0.02147~~	~~0.001~~	~~0.40153~~	~~46.57662~~	~~2.169381~~	~~0.0496~~	~~0.001~~	~~0.20506~~	~~0.00583~~	~~0.00039~~
~~18a~~	~~2664~~	~~22370~~	~~0.5327~~		~~0.1609~~	~~0.0052~~	~~0.02339~~	~~0.0011~~	~~0.45802~~	~~42.75331~~	~~2.01063~~	~~0.0505~~	~~0.0011~~	~~0.14338~~	~~0.00635~~	~~0.00043~~
~~19a~~	~~2083~~	~~20320~~	~~0.592~~		~~0.1689~~	~~0.0069~~	~~0.02245~~	~~0.0011~~	~~0.21911~~	~~44.54343~~	~~2.182529~~	~~0.0551~~	~~0.0021~~	~~0.36546~~	~~0.00612~~	~~0.00044~~
~~21a~~	~~1747~~	~~19410~~	~~0.709~~		~~0.1494~~	~~0.005~~	~~0.02221~~	~~0.0011~~	~~0.12929~~	~~45.02476~~	~~2.229952~~	~~0.0491~~	~~0.0012~~	~~0.16562~~	~~0.00583~~	~~0.0004~~
~~22a~~	~~5320~~	~~118000~~	~~0.448~~		~~0.1502~~	~~0.0056~~	~~0.02209~~	~~0.0011~~	~~0.44179~~	~~45.26935~~	~~2.254246~~	~~0.0497~~	~~0.0014~~	~~0.1861~~	~~0.00576~~	~~0.00042~~
~~23a~~	~~562~~	~~11350~~	~~0.547~~		~~0.1739~~	~~0.0096~~	~~0.02503~~	~~0.0013~~	~~0.33742~~	~~39.95206~~	~~2.075017~~	~~0.0514~~	~~0.0025~~	~~0.16474~~	~~0.00576~~	~~0.00056~~
28a	1197	697	2.93		0.1609	0.0053	0.0237	0.0011	0.37217	42.19409	1.958376	0.0496	0.0012	0.16404	0.00666	0.00049
30a	1427	1179	1.191		0.1612	0.0058	0.02422	0.0012	0.2985	41.28819	2.045658	0.0486	0.0014	0.23245	0.00664	0.00045
31a	1750	1795	0.861		0.1608	0.0054	0.02388	0.0012	0.46649	41.87605	2.104324	0.0487	0.0011	0.09649	0.00653	0.00045
~~32a~~	~~3590~~	~~1478~~	~~2.011~~		~~0.1664~~	~~0.0053~~	~~0.0239~~	~~0.0012~~	~~0.62178~~	~~41.841~~	~~2.100804~~	~~0.05047~~	~~0.00095~~	~~-0.015~~	~~0.00627~~	~~0.00043~~
33a	1773	1940	0.817		0.1603	0.0071	0.02295	0.0012	0.43364	43.57298	2.278326	0.0507	0.0018	0.1331	0.00609	0.00045
~~35a~~	~~5000~~	~~7550~~	~~0.5963~~		~~0.1577~~	~~0.0043~~	~~0.02325~~	~~0.0011~~	~~0.58177~~	~~43.01075~~	~~2.034917~~	~~0.04942~~	~~0.0007~~	~~0.10043~~	~~0.00644~~	~~0.00042~~
36a	1930	1360	1.442		0.1548	0.006	0.02247	0.0012	0.41036	44.50378	2.376704	0.0505	0.0015	0.26325	0.00634	0.00048
37a	1690	1460	1.101		0.173	0.0075	0.02483	0.0013	0.46883	40.27386	2.108579	0.051	0.0017	0.11887	0.00634	0.00049
~~38a~~	~~3200~~	~~3210~~	~~0.92~~		~~0.1602~~	~~0.0045~~	~~0.02362~~	~~0.0011~~	~~0.42706~~	~~42.337~~	~~1.971664~~	~~0.04974~~	~~0.00079~~	~~0.19957~~	~~0.00635~~	~~0.00042~~
41a	1463	1270	1.228		0.1687	0.0054	0.02416	0.0012	0.43084	41.39073	2.055831	0.0504	0.0011	0.09644	0.00643	0.00045
42a	462	523.7	0.962		0.1663	0.0076	0.02472	0.0012	0.19023	40.45307	1.963741	0.0486	0.0018	0.07031	0.00678	0.0005
43a	1352	1291	1.16		0.1644	0.0052	0.02402	0.0011	0.27313	41.63197	1.906543	0.0499	0.0012	0.28616	0.00646	0.00045
44a	1118	802	3.61		0.1649	0.0057	0.02387	0.0011	0.3096	41.89359	1.93058	0.0502	0.0013	0.16209	0.00698	0.00055
~~45a~~	~~4360~~	~~7690~~	~~0.606~~		~~0.1582~~	~~0.0044~~	~~0.02265~~	~~0.0011~~	~~0.52757~~	~~44.15011~~	~~2.144155~~	~~0.05045~~	~~0.00078~~	~~0.15947~~	~~0.00627~~	~~0.00041~~
~~47a~~	~~3852~~	~~5890~~	~~0.705~~	-	~~0.1579~~	~~0.0043~~	~~0.02275~~	~~0.0011~~	~~0.53644~~	~~43.95604~~	~~2.125347~~	~~0.0501~~	~~0.00071~~	~~0.19161~~	~~0.0064~~	~~0.00042~~

(continued)

	Ages (Ma)								Zircon radiation damage	
spot #	$^{207}Pb/^{235}U$	2SE	$^{206}Pb/^{238}U$	2SE	$^{207}Pb/^{206}Pb$	2SE	$^{208}Pb/^{232}Th$	2SE	D (Murakami et al. 1991)	Radiation damage stage (Nasdala et al. 2004)
~~01a~~	~~145~~	~~8~~	~~146~~	~~9~~	~~160~~	~~110~~	~~119~~	~~11~~	~~2.08854E+15~~	~~2~~
~~03a~~	~~152~~	~~6~~	~~149~~	~~8~~	~~179~~	~~69~~	~~129~~	~~10~~	~~1.61186E+15~~	~~2~~
~~06a~~	~~149~~	~~4~~	~~149~~	~~7~~	~~155~~	~~29~~	~~130~~	~~9~~	~~5.83405E+15~~	~~3~~
~~07a~~	~~150~~	~~4~~	~~149~~	~~7~~	~~176~~	~~49~~	~~130~~	~~9~~	~~2.97523E+15~~	~~2~~
08a	150	5	151	7	153	52	128	9	1.44846E+15	1
11a	157	4	154	7	200	39	134	10	1.4355E+15	1
~~12a~~	~~138~~	~~4~~	~~137~~	~~7~~	~~163~~	~~46~~	~~118~~	~~8~~	~~3.60799E+15~~	~~2~~
~~18a~~	~~151~~	~~5~~	~~149~~	~~7~~	~~203~~	~~48~~	~~128~~	~~9~~	~~3.88325E+15~~	~~2~~
~~19a~~	~~159~~	~~6~~	~~143~~	~~7~~	~~377~~	~~82~~	~~123~~	~~9~~	~~3.22597E+15~~	~~2~~
~~21a~~	~~141~~	~~4~~	~~142~~	~~7~~	~~130~~	~~52~~	~~118~~	~~8~~	~~2.9465E+15~~	~~2~~
~~22a~~	~~142~~	~~5~~	~~141~~	~~7~~	~~186~~	~~67~~	~~116~~	~~9~~	~~1.5329E+16~~	~~3~~
~~23a~~	~~162~~	~~8~~	~~159~~	~~8~~	~~220~~	~~110~~	~~116~~	~~11~~	~~1.69008E+15~~	~~2~~
28a	151	5	151	7	154	49	134	10	6.76127E+14	1
30a	152	5	154	7	110	59	134	9	8.63726E+14	1
31a	151	5	152	7	121	49	132	9	1.08634E+15	1
~~32a~~	~~156~~	~~5~~	~~152~~	~~7~~	~~201~~	~~41~~	~~126~~	~~9~~	~~1.96944E+15~~	~~2~~
33a	151	6	146	7	202	76	123	9	1.07036E+15	1
~~35a~~	~~149~~	~~4~~	~~148~~	~~7~~	~~158~~	~~31~~	~~130~~	~~9~~	~~3.29826E+15~~	~~2~~
36a	146	5	143	7	216	70	128	10	1.0578E+15	1
37a	162	7	158	8	224	74	128	10	1.05872E+15	1
~~38a~~	~~151~~	~~4~~	~~151~~	~~7~~	~~171~~	~~35~~	~~128~~	~~9~~	~~1.96477E+15~~	~~2~~
41a	158	5	154	7	199	47	130	9	8.92812E+14	1
42a	155	7	157	8	95	76	137	10	3.02398E+14	1
43a	154	5	153	7	168	51	130	9	8.33532E+14	1
44a	155	5	152	7	177	56	141	11	6.5349E+14	1
~~45a~~	~~149~~	~~4~~	~~144~~	~~7~~	~~202~~	~~35~~	~~126~~	~~8~~	~~2.92067E+15~~	~~2~~
~~47a~~	~~149~~	~~4~~	~~145~~	~~7~~	~~195~~	~~33~~	~~129~~	~~9~~	~~2.49714E+15~~	~~2~~
