# Supplementary material for: The Montecristo mining district, northern Chile: the relationship between vein-like magnetite-(apatite) and iron oxide-copper–gold deposits
Source: Miner Depos. 2023 Mar 28;58(6):1023–49. doi: 10.1007/s00126-023-01172-0 (PMC10329088; doi:10.1007/s00126-023-01172-0)
Supplement: Supplementary file 7 — Supplementary file7 (PDF 83 KB) [file 126_2023_1172_MOESM7_ESM.pdf]

ESM Table 7. Re-Os results on two molybdenite samples from the Abundancia deposit.

AIRIE Run #	Sample Name	Description	Re, ppm	±	^{187}Os, ppb	±	Os_{C}, ppb	±	Age, Ma	±	Sample wt (g)
MD-1870	MOC-18-07A	Slickenside fracture	19.85	0.63	34.01	0.19	0.0024	0.0003	163.4	5.3	0.15963
MD-1886	MOC-18-07A	Slickenside fracture (R)	23.138	0.042	39.395	0.015	0.0000	0.0004	162.38	0.60	0.05329
MD-1887	MOC-18-07G	Slickenside fracture	369.72	0.60	588.38	0.20	0.022	0.005	151.79	0.55	0.02172

Re-Os determinations used a Carius tube dissolution with a mixed double-Os spike (Markey et al. 2003).

All data reported at 2-sigma uncertainty, and error on ages includes the ^{187}Re decay constant uncertainty (λ)

R = Rerun using the same mineral separate as MD-1870 to correct for spiking; age uncertainty is reduced.

Decay constant of Smoliar et al (1996) used for age calculations.

Os_{C} = Common (initial) osmium; Assumed Os initial ratio = 0.2.

Re blank = 2.13 ± 0.06 pg, Os blank = 0.099 ± 0.008 pg with $^{187}Os/^{188}Os$ = 0.350 ± 0.023

Blanks and common Os present are both insignificant to the Re-Os age calculations.
